# Interaction between influenza A virus nucleoprotein and PB2 cap-binding domain is mediated by RNA

**DOI:** 10.1371/journal.pone.0239899

**Published:** 2020-09-28

**Authors:** Wun-Chung Szeto, Ho-Pan Hsia, Yun-Sang Tang, Pang-Chui Shaw

**Affiliations:** Centre for Protein Science and Crystallography, School of Life Sciences, The Chinese University of Hong Kong, Hong Kong, China; Consejo Superior de Investigaciones Cientificas, SPAIN

## Abstract

Influenza A virus controls replication and transcription of its genome through the tight regulation of interaction between the ribonucleoprotein (RNP) complex subunits. The helical scaffold of RNP is maintained by nucleoprotein (NP). Previous studies have revealed that NP interacts with both PB2 N-terminal and C-terminal regions, with both regions sharing similar affinity to NP as revealed in co-immunoprecipitation assay. Our work here suggests that the interaction between NP and PB2 N-terminal region lies in the cap-binding domain (residue 320–483). By co-immunoprecipitation assay, the interaction was found to involve RNA. On the other hand, the cap-binding activity was not essential in the interaction. As shown by the NHS pull-down assay, a specific RNA sequence was not required. Among the cap-binding domain, residues K331 and R332 of PB2 play a role in RNP function so that polymerase activity was reduced when these residues were mutated, while K331 was found to be more crucial in the NP interaction. Collectively, our findings suggest a new binding mode between NP and PB2 which was mediated by RNA, and such interaction may provide a novel interacting site for influenza drug development.

## Introduction

Influenza virus is one of the major causes of contagious respiratory disease. Annual influenza epidemics can claim up to 650,000 lives every year, causing a heavy burden to the healthcare system. Genome of influenza A virus consists eight segments of single stranded, negative sense viral RNA (vRNA) which is packaged into a rod-shape viral ribonucleoprotein (vRNP) complex scaffold [1–3]. This complex is assembled by multiple nucleoproteins (NPs) and a heterotrimeric RNA-dependent RNA polymerase (RdRP). Transcription and replication of the vRNA are sophisticatedly controlled by the interaction between the RdRP subunits and NP [2, 4]. NP oligomer is arranged in a double helical conformation to coat vRNA while the RdRP binds the 3’ and 5’ termini of the vRNA [1, 2, 5].

RdRP of influenza A virus is composed of subunits PA, PB1 and PB2. PB1 forms the core and is wrapped by PA C-terminal domain and PB2 N-terminal domain [6, 7]. The catalytic site of the RNA polymerase resides in PB1 which contains the finger, palm and thumb subdomains. N-terminal domain of PA subunit is responsible for endonuclease activity [8], and it is connected to a large C-terminal domain by a linker [2, 6, 7]. Other than forming the polymerase core with PB1, PB2 subunit consists of several domains serving multiple purposes. Host range determination of influenza A virus is achieved by a well-studied residue at position 627 of PB2, which gives the name of PB2 ‘627-domain’ (amino acids 538–693) [9]. Polymerase variants without ‘627-domain’ maintained its functions *in vitro* but not in cells [10], suggesting its role in host cell interaction. The cap-binding domain of PB2 (aa 320–483) is involved in the cap-snatching process. It binds host 5’ capped RNAs for later cleavage by the PA endonuclease to produce capped RNA fragment used as primer for the initiation of transcription of viral genome [6, 11].

NP is the most abundant protein in the RNP complex, NP monomers are linked together by insertion the tail-loop of one NP protomer to its neighbouring molecule. NP oligomerization provides a RNA-binding surface for vRNA and complementary RNA (cRNA) [1, 12, 13]. Previous studies show that NP interacts with all three polymerase subunits [14–17]. Earlier studies suggested that NP served as a switch between transcription and replication [17–19], which was challenged by a more recent evidence showing that NP is recruited as an elongation factor during vRNA and cRNA synthesis but not as a switch [20].

X-ray crystallography and cryo-electron microscopy have demonstrated inter-subunit interactions within the RdRP to high resolution [5, 6, 21, 22]. However, NP-PB2 interaction is to-date not clearly defined, Three independent regions of NP (aa 1–161, 255–340, 340–465) were found to bind PB2 [14] while two fragments of PB2 (aa 1–269 and 580–683) were shown to interact with NP [16], D605 of PB2 ‘627-domain’ was later mapped to interact with NP where V606 of PB2 also showed a weak binding with NP [23]. On the other hand how the N-terminal fragment of PB2 interacts with NP is not clear. *In silico* docking suggested the interaction occurred in the first 24 amino acids [24], and *in vitro* study claimed PB2 interacts with NP via its N-terminal 269 residues [16]. This report aims at revealing how NP interacts with the N-terminal region of PB2.

## Results

### Cap-binding domain of PB2 interacts with NP

**Fig 1:**
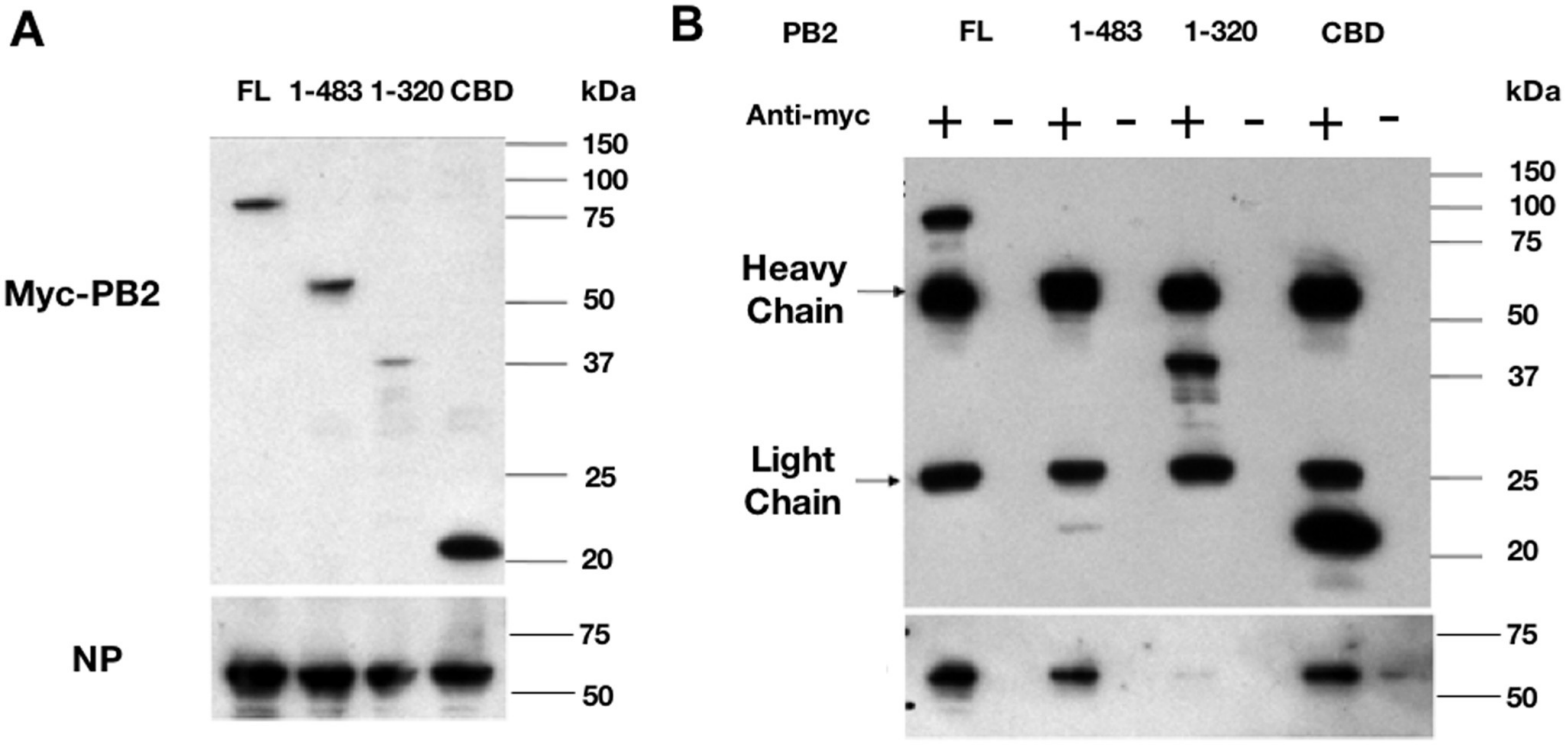
Co-immunoprecipitation analysis of NP and PB2 N-terminal fragments. (A) Expression of NP, PB2 and PB2 truncation mutants. Full length PB2 and three truncated constructs (aa 1–483, 1–320 and CBD) of myc-tagged PB2 were co-transfected with NP into HEK 293 cells. Total cell lysate was collected after 48-hour incubation to detect NP and myc-tagged PB2 fragments expression by Western blotting analysis. (B) Co-immunoprecipitation of NP and PB2 or PB2 fragments. Co-immunoprecipitation was performed with overnight incubation of anti-myc antibody with cell lysate, protein A agarose was used to precipitate the antibody. Amount of NP precipitated was detected by anti-NP serum, and the amount of myc-tagged PB2 and PB2 fragments were detected by anti-myc antibody. FL: Full-length PB2. CBD: Cap-binding domain.

We first set forth to identify and confirm the regions on PB2 interacting with NP using a co-immunoprecipitation assay. PB2 truncation mutants were generated by deletion mutagenesis and tagged with myc peptide. Myc-tagged PB2 deletion mutants and NP were co-expressed in HEK 293 cells by transfection. Consistent with previous findings, full-length PB2 could efficiently precipitate NP and PB2 fragment 1–483 could precipitate comparable amount of NP as full-length (Fig 1B, lanes 1–4). This fragment was then further divided into two regions, PB2 fragment 1–320 and cap-binding domain (aa 320–483), to locate the NP-binding region. Fragment 1–320 interacted very weakly with NP despite this fragment contained the N-terminal 269 residues which was proposed for NP binding [16]. The cap-binding domain (CBD) precipitated significant amount of NP (Fig 1B, lane 5–8). Which was comparable to the amount precipitated by full-length PB2 (Fig 1B). Hence, the major NP-binding region resided within the PB2 N-terminal in the cap-binding domain (aa 320–483).

### Interaction between PB2 cap-binding domain and NP is mediated by RNA

**Fig 2:**
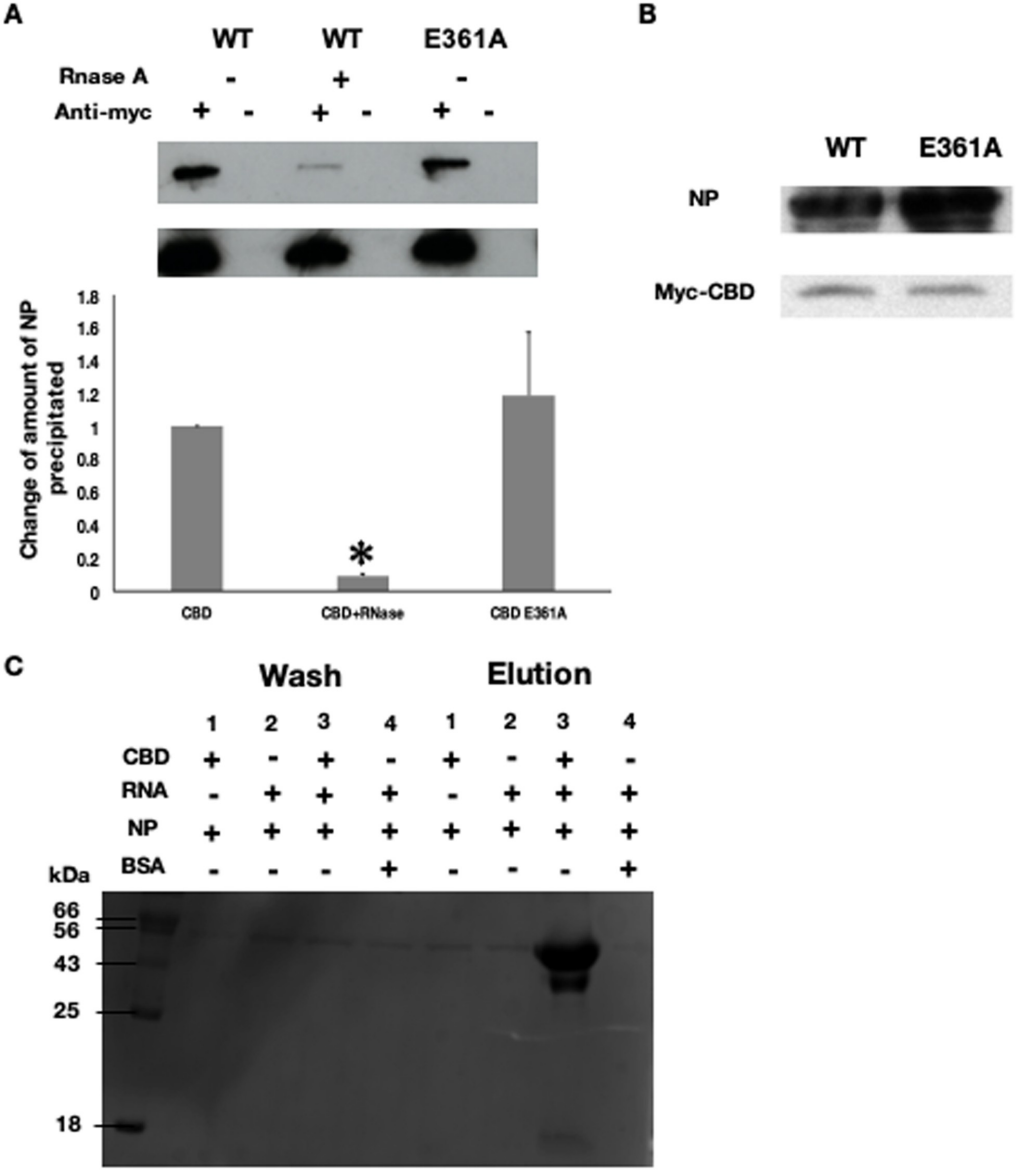
Characterization of cap-binding domain-NP interaction. (A) Co-immunoprecipitation of NP and myc-tagged PB2 cap-binding domain with and without addition of RNase A. The bar represents the mean ratio ± standard errors from two independent experiments. *The p-values are smaller or equal to 0.05 in two-tailed Student’s *t*-test. (B) Expression of NP, PB2 cap-binding domain and E361A variant. Wild-type cap-binding domain (CBD WT) or E361A mutant was co-transfected with NP into HEK 293 cells. Protein expression was detected by anti-NP serum and anti-myc antibody. (C) Pull-down assay investigating NP-PB2 cap-binding domain binding. NHS-activated sepharose was used to immobilize 50μM PB2 CBD, BSA was immobilized as control (sample 4). Purified NP and 24-nt 2’-O-methylated RNA were added accordingly, the mixtures were washed extensively after 1 hour incubation. 1.5M NaCl was used to elute the protein captured by CBD. Samples of wash and elution fractions were separated by 12% SDS-PAGE and detected by coomassie blue staining. WT: Wild type.

Cap-binding domain of PB2 was reported for its cap-snatching activity during transcription of the influenza viral genome [9, 24]. This domain is responsible for binding the 5’ methyl cap of the host mRNA and thus facilitating cleavage by PA endonuclease. After cleavage, the domain rotates *in situ* to direct cleaved mRNA to the PB1 active site for priming transcription reaction. RNA binding activity of the CBD has been reported and at the same time, NP also has a high affinity to RNA [25, 26]. We then therefore investigate whether RNA play any roles in mediating NP-CBD interaction. To this end we added RNase A into the cell lysate lysate to remove endogenous RNA before co-immmunoprecipitation. After RNA cleavage, cap-binding domain became unable to pull down NP (Fig 2A). This observation suggested the requirement of RNA in the interaction between NP and PB2 cap-binding domain. We then tested whether the interaction must be mediated by a capped, primer-like RNA. Thus we co-expressed a cap-binding domain variant E361A with NP in HEK 293 cells and repeated the co-immunoprecipitation analysis. E361A mutation was previously reported to reduce the affinity of cap-binding domain to 7-methylguanosine 5’-triphosphate (m^7^GTP), a structural analogue of eukaryotic cellular mRNA caps, by more than 8 folds [27]. Comparable amount of NP was co-immunoprecipitated by E361A and wild-type PB2 (Fig 2A), suggesting the cap of RNA is not needed for the interaction. We also investigated whether the sequence of RNA is important for the interaction. NP and PB2 cap-binding domain were individually expressed in *E*. *coli* and purified for *in vitro* binding test. A short synthetic 2’-O-methylated RNA of 24 nucleotides long was added. NP was detected in the elution only when the synthetic RNA was added (Fig 2C), suggesting that NP-PB2 cap-binding domain interaction is independent of the sequence of RNA. Taken together, our results showed that NP-CBD interaction requires the presence of RNA but not a 5’methyl cap or a specific sequence.

### Alanine substitution of basic residues of PB2 cap-binding domain reduces RNP activity and NP-binding

**Fig 3:**
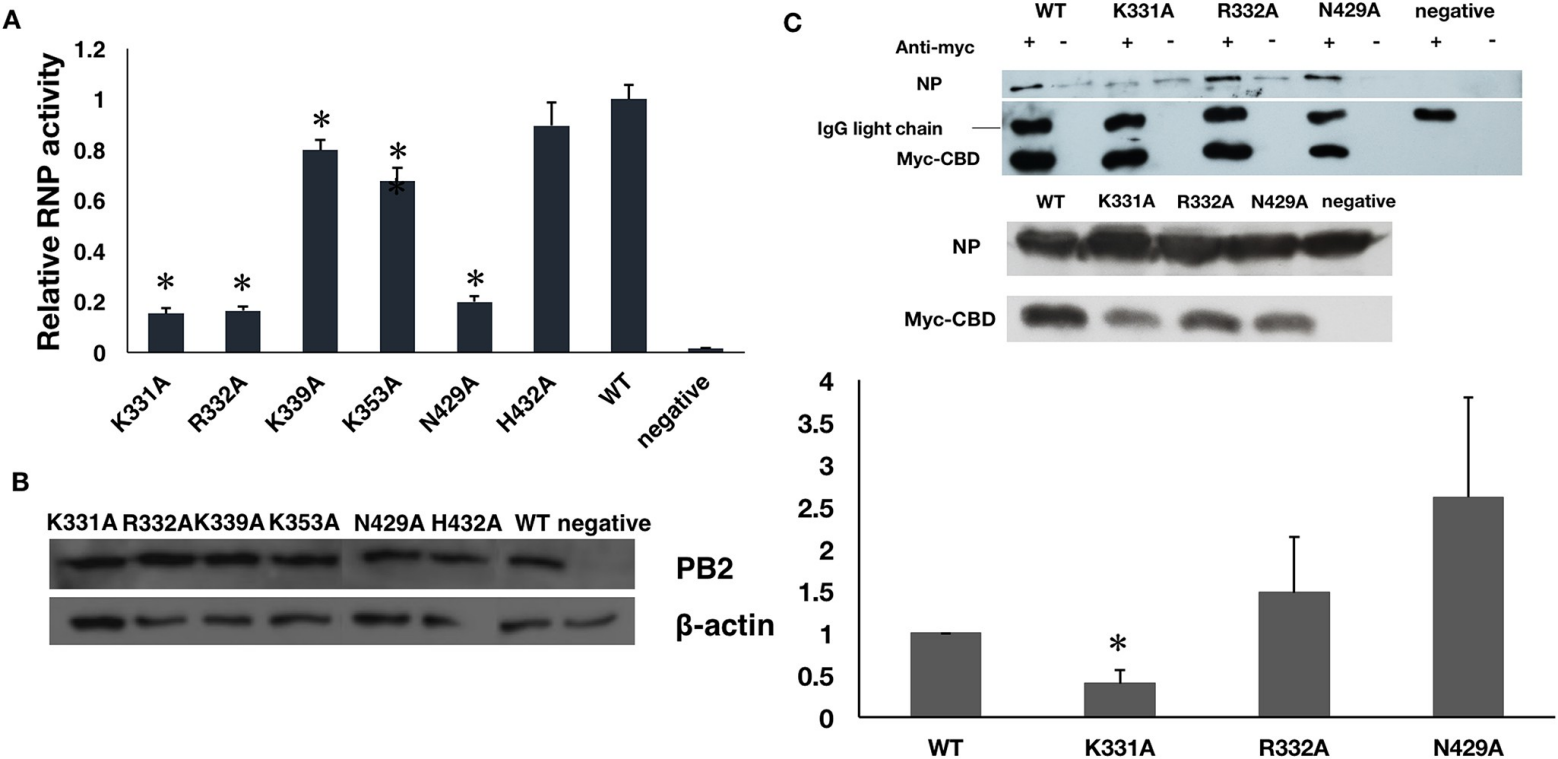
Effect of PB2 variants on RNP activity and NP-binding. (A) Polymerase activity measured by luciferase reporter assay. Plasmids encoding wild-type or mutant PB2, PA, PB1, NP (ie. pcDNA-PA, pcDNA-PB1, pcDNA-PB2 and pcDNA-NP) were co-transfected with a plasmid expressing luciferase reporter gene under viral promoter control, pPOLI-Luc-RT and a plasmid expressing GFP, pEGFP were transfected to HEK 293T, pcDNA3 was used to replace pcDNA-PB2 as negative control. The overall polymerase activity is represented by a ratio of luminescence signal to GFP signal. The activity of polymerase with wild-type PB2 was set to 1. The bar represents the mean ratio ± standard errors from three independent experiments. *The p-values are smaller or equal to 0.05 in two-tailed Student’s *t*-test. (B) Protein expression of PB2 and mutants. Level of PB2 expression of each mutant was detected by anti-PB2 polyclonal antibodies in Western blotting with ß-actin as loading control. (C) Co-immunoprecipitation of NP and cap-binding domain. Myc-tagged PB2 cap-binding domain and variants were co-transfected and expressed with NP in HEK 293 cells. Myc-tagged empty vector was used as negative control. Co-immunoprecipitation was performed after 48-hour incubation, cell lysates were incubated overnight with or without anti-myc antibody. Expression levels of myc-tagged PB2 cap-binding domain and variants were detected by anti-myc antibody, while anti-NP serum was used to detect NP expression level in the cells. Amounts of NP precipitated by cap-binding domain variants in Co-immunoprecipitation were compared to that precipitated from the wild-type. The bar represents the mean ratio ± standard errors from three independent experiments. *The p-values are smaller or equal to 0.05 in two-tailed Student’s *t*-test.

We then attempted to identify residues on PB2 CBD which may be important in mediating NP binding. As RNA is involved in the interaction, basic residues around the cap binding site of PB2 cap-binding domain may be involved in binding RNA and NP. Residue K331, R332, K339, K353, N429 and H432 on the surface of the cap-binding pocket were found to bind or are in close proximity of the capped RNA primer of a transcribing polymerase [25]. By luciferase reporter assay PB2 mutants K331A, R332A and N429A reduced polymerase activity to 10–20% of normal level, while K339, K353 and H432 only showed mild effects (Fig 3A). We repeated co-immunoprecipitation assay on K331A, R332A and N429A, to confirm whether NP binding was affected. Mutant K331A exhibited a drop of NP precipitation in assay while such effect was not observed in R332A and N429A (Fig 3C).

**Fig 4:**
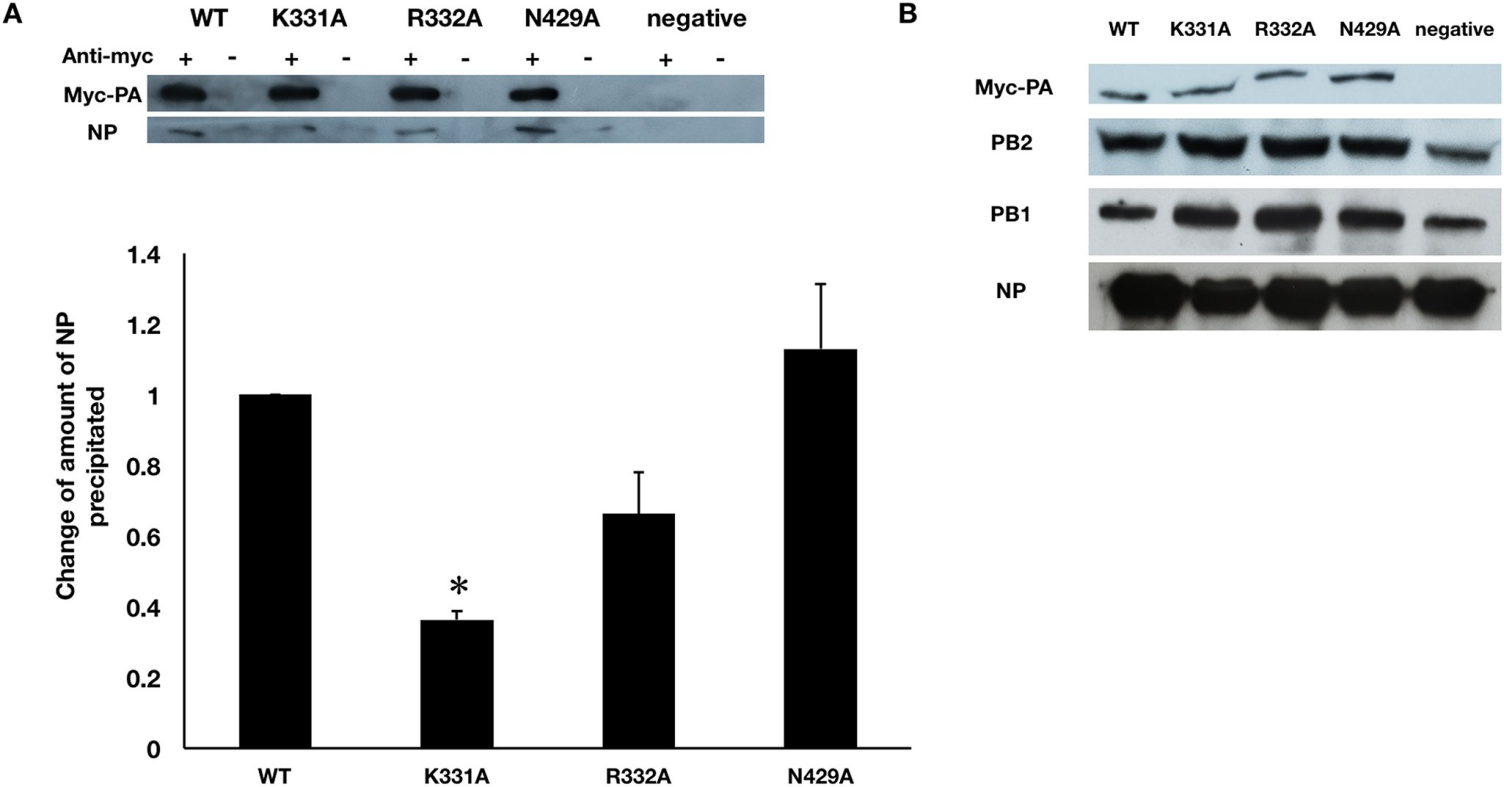
Effects of PB2 variants on NP-polymerase interaction. (A) Co-immunoprecipitaion of NP-polymerase. Plasmids encoding myc-tagged PA, PB1, PB2 or its variants, pPOLI-NA-RT were co-transfected with NP into HEK 293T cells, myc-tagged empty vector was used as negative control. Cells were harvested after 48-hour incubation. Cell lysates were incubated overnight with or without anti-myc antibody. Amount of NP co-immunoprecipitated with PB2 variants were compared to that precipitated from the wild-type. The bar represents the mean ratio ± standard errors from three independent experiments. *The p-values are smaller or equal to 0.05 in two-tailed Student’s *t*-test. (B) Expression of RNP subunits. Levels of expression of RNP subunits were detected by corresponding anti-bodies.

We postulated that RNA-mediated NP-CBD interaction could be important for the formation of a functional vRNP. As such, we attempted to reconstitute the RNP complex with a replication mutant PB1 D446Y [28]. Employing PB1 D446Y mutant instead of wild-type PB1 eliminated any possible effects due to variation in the replication efficiency of the RdRP and allowed us to compare vRNP formation on the basis of a fixed amount of vRNA. We co-transfected into HEK 293T cells plasmids expressing PB1-D446Y, PB2 (wild-type or mutants), NP, myc-tagged PA and a pPol-NA-RT plasmid which provided the genomic vRNA of segment 6 serving as template. In the context of PB2 K331A, about 50% less NP was co-immunoprecipitated, compared to the wild-type PB2, while such decrease was not observed by PB2 R332A or N429A mutations (Fig 4A). These results suggested a disruption of RNP formation when K331 was mutated. Hence K331A mutation of PB2 reduces polymerase activity by disrupting RNP formation.

## Discussion

**Fig 5:**
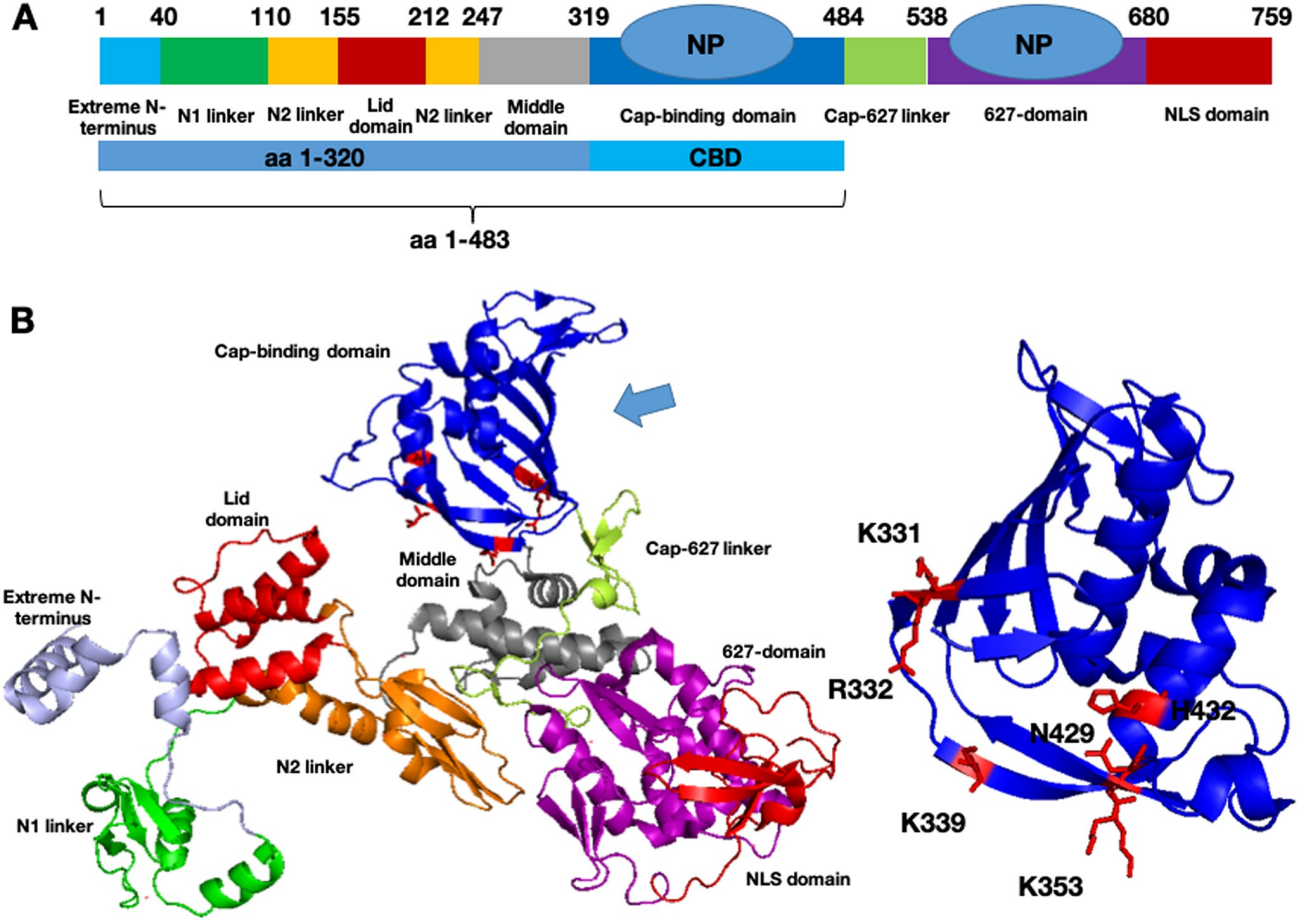
Structure of PB2 and PB2 cap-binding domain. (A) Schematic of multiple domains of PB2. Schematic of NP was added to indicate NP binding site. Studied regions (aa 1–320, CBD and aa 1–483) were highlighted. (B) (Left) Structure of PB2 from crystal structure of influenza polymerase (PDB ID: 4WSB). Structure showed that domains of PB2 are joined by several flexible linkers and suggested a high degree of flexibility. Studied region was indicated by arrow and studied amino acids of the cap-binding domain were indicated in red. (Right) Structure of PB2 cap-binding domain. Basic residues around the exit of cap-binding pocket (K331, R332, K339, K353, N429, H432) were indicated in red. (PDB ID: 4CB4).

The current study reveals a novel interaction between NP and PB2 cap-binding domain. In a previous study, residue 1–320 of PB2 expressed in *Xenopus* oocytes was found to bind GST-NP in GST pulldown assay [16]. However in this study, we showed that this PB2 fragment precipitated NP very weakly in co-immunoprecipitation experiment (Fig 1B). In contrast, the interaction between PB2 cap-binding domain and NP was much stronger (Fig 1B). Focusing on the CBD, we further defined important residues for NP-binding and delineated the underlying mechanism. In fact our result differs from the finding of Poole et al. who showed no interaction between GST-NP and PB2 aa 283–590 [16] which contained the CBD residues. This discrepancy is probably due to the fact that aa 283–590 of PB2 is not a well-defined region. According to the recently published structures, the PB2 fragment used by Poole et al. [16] comprises part of the mid domain (aa 247–319), the cap-binding domain (aa 320–483), the cap-627 linker (aa 484–538) and part of the 627 domain (aa 539–680) (Fig 5A and 5B). As a result, this multi-domain fragment might not be properly folded for the PB2-NP interaction.

In our study, mutating basic residues K331, R332 and N429 to alanine on PB2 CBD led to a reduced RNP activity, which implied a role of these residues in replication or transcription of influenza viral genome. In fact, Hara et al. also showed reduced polymerase activity with K331A/R332A double mutations on PB2 [29]. However the authors did not demonstrate the cause of this reduction in activity. Our work shows that K331 reduced NP binding upon mutation to alanine, disrupted NP-PB2 cap-binding domain interaction and also the formation of the RNP complex. As such, we have illustrated the importance of NP-CBD interaction in the RNP formation which is likely mediated by K331 in PB2.

In our previous work [23, 30] we identified that PB2 627-domain interacted with NP and the interaction was mediated by PB2 D605. Here in this work we showed that PB2 also interacts with NP via its cap-binding domain, mediated by RNA. Since PB2 ‘627-domain’ adopts radically different conformations during RNA synthesis [31], NP-‘627-domain’ interaction could be important in supporting the dynamic replication and transcription activities. Recent structural work provided a ‘sliding’ model that the transcribing polymerase slides on the chain of NP oligomers [5]. This ‘sliding’ process may likely be supported by the flexible and protruding ‘627-domain’ of PB2 as the radically rearranging ‘627-domain’ interacts with NP oligomers and may drive the polymerase to slide along the chain of NP oligomers. The cap-binding domain on the other hand is capable of *in situ* rotation during the transcription process which is relatively less flexible. It is possible that the cap-binding domain anchors both NP and the vRNA or cRNA during their formation. Our observation that K331A disrupted vRNP formation provides support to this postulation. Taken together, our work provides a more in-depth understanding on the different NP-PB2 interaction for the function of the RNP.

The interaction between NP and PB2 cap-binding domain contributes to the transcription activity of RNP, and is a potential drug target for developing anti-influenza agents.

## Materials and methods

### Biological materials

Virus strain used in this study was A/Hong Kong/156/97. HEK 293 cells (Catalogue number: CRL-1573) and HEK 293T cells (Catalogue number: CRL-11268) were obtained from ATCC, Manassas, VA, USA and maintained under 37°C, 5% CO_2_ in Dulbecco’s Modified Eagle’s Medium (DMEM) (Life Technologies) with 10% Fetal Bovine Serum (Life Technologies). Anti-NP serum was prepared from NP immunized rabbits by Guangdong Medical Laboratory Animal Centre, specificity of the anti-serum was tested (S1 Fig). Anti-myc antibodies were purchased from Cell Signaling (Catalogue number: 2276S). Anti-PB1 antibodies were obtained from bei resources (Catalogue number: NR-31691) and anti-PB2 antibodies were purchased from GeneTex (Catalogue number: GTX125926-S). Plasmids pcDNA-PA, pcDNA-PB1, pcDNA-PB2 and pcDNA-NP encoding sequences of influenza strain A/Hong Kong/156/97 RNP subunits were kind gifts from Prof. Ervin Fodor (University of Oxford) and was described previously [26]. While pPOLI-Luc-RT, pEGFP and pPOLI-NA-RT were received from Prof. L.L.M Poon (The University of Hong Kong). Plasmids encoding myc-tagged PA, PB2 were generated by inserting the corresponding sequences into vector pcDNA3.1/myc-his A (Invitrogen) by T4 DNA ligase (ThermoFisher) after the inserts and vectors were digested by restriction enzymes (NEB). PB2 fragment sequences were generated by PCR with forward and reverse primers amplifying the fragment regions. Sequences with point mutation were produced by overlap extension PCR. Sequences were also inserted into pcDNA3 (Invitrogen) or pcDNA3.1/myc-his A. Plasmids for expression of NP and PB2 cap-binding domain in *E*. *coli* were generated by inserting the coding sequences into pET-28a (Invitrogen) by the mentioned method.

### Expression and purification of NP and PB2 cap-binding domain variant

Plasmids were transformed into BL21 (DE3) pLysS competent E. coli. 0.4 mM IPTG was added to the bacterial cultures when OD_600_ of the cultures reached 0.6–0.8 for induction of protein expression. Bacterial cultures were incubated overnight at 18°C and 21°C for PB2 cap-binding domain and NP respectively. Proteins were purified as described previously [26, 32]. Cells were harvested and lysed by sonication, His-tagged proteins expressed were captured by Ni-sepharose (GE Healthcare) and further purified by size exclusion chromatography.

### Co-immunoprecipitation of NP-PB2 cap-binding domain and NP-polymerase

1x10^6^ HEK 293 cells were seeded in 6cm dish 24 hours before transfection. 4μg of myc-tagged wild-type or PB2 fragment encoding plasmids and 1μg of pcDNA-NP were transfected with lipofectamine 2000 (Life Technologies) according to the manufacturer’s protocol. Cells were harvested and lysed by sonication in IP buffer (50mM Tris pH 7.6, 150mM NaCl, 1mM EDTA, 1% Triton X-100) 48 hours after transfection. Cell lysate was centrifuged at 18000g, 4°C for 10 minutes to remove cell debris. The supernatant was incubated with anti-myc antibodies (Cell signaling) and 10U RNase A (Sigma Aldrich) when appropriate for overnight at 4°C. Protein A agarose (Sigma Aldrich) was added to each sample for further incubation at 4°C for 1.5 hours. The beads were washed with IP buffer before boiled in SDS loading buffer and analysed by Western blot. For co-immunoprecipitation of RNP complex, 8x10^5^ HEK 293T cells were seeded in 6 well plate 24 hour before transfection. 1 μg of each plasmid encoding myc-tagged PA, untagged PB1 D446Y mutant, PB2 wild-type or mutant, NP were transfected into the cells with pPOLI-NA-RT. Cells were harvested and analysed as mentioned above.

### *In vitro* binding test of purified NP and PB2 cap-binding domain

Purified PB2 cap-binding domain was immobilized on NHS-activated sepharose (GE Healthcare) according to the manufacturer’s protocol, BSA was immobilized in negative control. 50μM wild-type purified NP and 10μM 24-nucleotide long 2’-O-methylated RNA (RiboBio) were added accordingly for 1-hour incubation in buffer (50mM Tris–HCl pH 8, 300mM NaCl). The beads were then washed with the buffer and eluted in 1.5M NaCl. The eluted fractions were analysed in 12% SDS-PAGE, proteins were stained and visualized by coomassie blue.

### Influenza polymerase activity analysis by luciferase reporter assay

0.125 μg each of pcDNA-PA, pcDNA-PB1, pcDNA-PB2 or mutants and pcDNA-NP, pPOLI-Luc-RT and pEGFP plasmids were diluted to 12.5 μl in OptiMEM (Life Technologies) and added to another 12.5 μl OptiMEM with 1.05 μl of Lipofectamine 2000. pcDNA3 empty vector was used to replace as pcDNA-PB2 negative control. The transfection mixture was incubated for 20 min in a 96-well plate before adding 75 μl 1x10^5^ HEK 293T cell suspension in DMEM to the well. At 48 h post-transfection, GFP fluorescent signal was measured by microplate reader (BMG labtech) at excitation wavelength of 470nm and emission wavelength of 515nm. Subsequently, cells were lysed by Steady-Glo assay reagent (Promega) for 5 min before luminescence signal was measured. The polymerase activity was expressed as a ratio of luminescence to GFP signal.

## Supporting information

S1 File (PDF)

S1 Fig Specificity and affinity of anti-NP serum. Plasmid encoding myc-tagged NP was transfected into HEK 293 cells, cell lysate was obtained after 48 hour incubation. Expressed proteins were detected by anti-myc monoclonal antibody and anti-NP serum respectively by Western blot.
(PDF)

## References

[1] Arranz R, Coloma R, Chichón FJ, Conesa JJ, Carrascosa JL, Valpuesta JM, et al 2012 The Structure of Native Influenza Virion Ribonucleoproteins. Science: 338: 1634–1637. 10.1126/science.1228172 23180776

[2] Lo CY, Tang YS, Shaw PC. 2018 Structure and Function of Influenza Virus Ribonucleoprotein In: Harris J, Bhella D, editors. Virus Protein and Nucleoprotein Complexes. Subcellular Biochemistry, vol 88: 95–28. 10.1007/978-981-10-8456-0_5 29900494

[3] Palese P. 1977 The genes of influenza virus. Cell 10:1–10. 10.1016/0092-8674(77)90133-7 837439

[4] Eisfeld A, Neumann G, Kawaoka Y. 2015 At the centre: influenza A virus ribonucleoproteins. Nat Rev Microbiol 13: 28–41. 10.1038/nrmicro3367 25417656 PMC5619696

[5] Coloma R, Arranz R, de la Rosa-Trevín JM, Sorzano COS, Munier S, Clero D, et al 2020 Structural insights into influenza A virus ribonucleoproteins reveal a processive helical track as transcription mechanism. Nat Microbiol 5: 727–734 10.1038/s41564-020-0675-3 32152587

[6] Pflug A, Guilligay D, Reich S, Cusack S. 2014 Structure of influenza A polymerase bound to the viral RNA promoter. Nature 516: 355–360. 10.1038/nature14008 25409142

[7] Te Velthuis AJ, Fodor E. 2016 Influenza virus RNA polymerase: insights into the mechanisms of viral RNA synthesis. Nat Rev Microbiol 14: 479–493. 10.1038/nrmicro.2016.87 27396566 PMC4966622

[8] Dias A, Bouvier D, Crépin T, McCathay A, Hart DJ, Baudin F, et al 2009 The cap-snatching endonuclease of influenza virus polymerase resides in the PA subunit. Nature 458: 914–918. 10.1038/nature07745 19194459

[9] Tarendeau F, Crepin T, Guilligay D, Ruigrok RWH, Cusack S, Hart DJ. 2008 Host determinant residue lysine 627 lies on the surface of a discrete, folded domain of influenza virus polymerase PB2 subunit. PLoS Pathog 4:e1000136 10.1371/journal.ppat.1000136 18769709 PMC2515345

[10] Nilsson BE, Te Velthuis AJ, Fodor E. 2017 Role of the PB2 627 Domain in Influenza A Virus Polymerase Function. J Virol 91: e02467–16. 10.1128/JVI.02467-16 28122973 PMC5355620

[11] Pflug A, Lukarska M, Resa-Infante P, Reich S, Cusack S. 2017 Structural insights into RNA synthesis by the influenza virus transcription-replication machine, Virus Res 234: 103–117. 10.1016/j.virusres.2017.01.013 28115197

[12] Moeller A, Kirchdoerfer R, Potter C, Carragher B, Wilson I. 2012 Organization of the influenza virus replication machinery. Science 338: 1631–4. 10.1126/science.1227270 23180774 PMC3578580

[13] York A, Hengrung N, Vreede F, Huiskonen J, Fodor E. 2013 Isolation and characterization of the positive-sense replicative intermediate of a negative-strand RNA virus. Proc Natl Acad Sci U S A, 110: E4238–E4245. 10.1073/pnas.1315068110 24145413 PMC3831450

[14] Biswas SK, Boutz PL, Nayak DP. 1998 Influenza virus nucleoprotein interacts with influenza virus polymerase proteins. J Virol 72:5493–5501. 10.1128/JVI.72.7.5493-5501.1998 9621005 PMC110190

[15] Portela A, Digard P. 2002 The influenza virus nucleoprotein: a multifunctional RNA-binding protein pivotal to virus replication. J Gen Virol 83:723–734. 10.1099/0022-1317-83-4-723 11907320

[16] Poole E, Elton D, Medcalf L, Digard P. 2004 Functional domains of the influenza A virus PB2 protein: identification of NP- and PB1-binding sites. Virology 321:120–133. 10.1016/j.virol.2003.12.022 15033571

[17] Vidic J, Noiray M, Bagchi A, Slama-Schwok A. 2016 Identification of a Novel Complex between the Nucleoprotein and PA(1–27) of Influenza A Virus Polymerase. Biochemistry 55:4259–4262. 10.1021/acs.biochem.6b00514 27431776

[18] Beaton AR, Krug RM. 1986 Transcription antitermination during influenza viral template RNA synthesis requires the nucleocapsid protein and the absence of a 5’ capped end. Proc Natl Acad Sci U S A 83:6282–6286. 10.1073/pnas.83.17.6282 3462695 PMC386487

[19] Shapiro GI, Krug RM. 1988 Influenza virus RNA replication in vitro: synthesis of viral template RNAs and virion RNAs in the absence of an added primer. J Virol 62:2285–2290. 10.1128/JVI.62.7.2285-2290.1988 2453679 PMC253375

[20] Turrell L, Lyall JW, Tiley LS, Fodor E, Vreede FT. 2013 The role and assembly mechanism of nucleoprotein in influenza A virus ribonucleoprotein complexes. Nat Commun 4:1591 10.1038/ncomms2589 23481399 PMC4168216

[21] Kouba T, Drncová P, Cusack S. 2019 Structural snapshots of actively transcribing influenza polymerase. Nat Struct Mol Biol 26: 460–470. 10.1038/s41594-019-0232-z 31160782 PMC7610713

[22] Wandzik JM, Kouba T, Karuppasamy M, Pflug A, Drncova P, Provaznik J, et al 2020 A Structure-Based Model for the Complete Transcription Cycle of Influenza Polymerase. Cell 181(4): 877–93. 10.1016/j.cell.2020.03.061 32304664

[23] Hsia HP, Yang YH, Szeto WC, Nilsson BE, Lo CY, Ng AKL, et al 2018 Amino acid substitutions affecting aspartic acid 605 and valine 606 decrease the interaction strength between the influenza virus RNA polymerase PB2 '627' domain and the viral nucleoprotein. PLoS One 13(1): e0191226 10.1371/journal.pone.0191226 29338047 PMC5770049

[24] Reich S, Guilligay D, Pflug A, Malet H, Berger I, Crépin T, et al 2014 Structural insight into cap-snatching and RNA synthesis by influenza polymerase. Nature 516:361–366. 10.1038/nature14009 25409151

[25] Pflug A, Gaudon S, Resa-Infante P, Lethier M, Reich S, Schulze WM, et al 2018 Capped RNA primer binding to influenza polymerase and implications for the mechanism of cap-binding inhibitors, Nucleic Acids Res 46(2): 956–971. 10.1093/nar/gkx1210 29202182 PMC5778463

[26] Ak Ng, Zhang H, Tan K, Li Z, Liu JH, Chan PK, et al 2008 Structure of the Influenza Virus A H5N1 Nucleoprotein: Implications for RNA Binding, Oligomerization, and Vaccine Design. FASEB J 22: 3638–3647. 10.1096/fj.08-112110 18614582 PMC2537428

[27] Guilligay D, Tarendeau F, Resa-Infante P, Coloma R, Crépin T, Sehr P, et al 2008 The structural basis for cap binding by influenza virus polymerase subunit PB2. Nat Struct Mol Biol 15(5): 500–506. 10.1038/nsmb.1421 18454157

[28] Cauldwell AV, Moncorgé O, Barclay WS. 2013 Unstable Polymerase-Nucleoprotein Interaction Is Not Responsible for Avian Influenza Virus Polymerase Restriction in Human Cells. J Virol 87(2): 1278–1284. 10.1128/JVI.02597-12 23115299 PMC3554100

[29] Hara K, Kashiwagi T, Hamada N, Watanabe H. 2017 Basic amino acids in the N-terminal half of the PB2 subunit of influenza virus RNA polymerase are involved in both transcription and replication. J Gen Virol, 98(5): 900–905. 10.1099/jgv.0.000750 28530165

[30] Ng AK, Chan WH, Choi ST, Lam MK, Lau KF, Chan PK, et al 2012 Influenza polymerase activity correlates with the strength of interaction between nucleoprotein and PB2 through the host-specific residue K/E627. PloS One 7(5): e36415 10.1371/journal.pone.0036415 22570712 PMC3343083

[31] Thierry E, Guilligay D, Kosinski J, Bock T, Gaudon S, Round A, et al 2016 Influenza Polymerase Can Adopt an Alternative Configuration Involving a Radical Repacking of PB2 Domains. Mol Cell 61(1): 125–137. 10.1016/j.molcel.2015.11.016 26711008 PMC4712189

[32] Liu Y, Qin K, Meng G, Zhang J, Zhou J, Zhao G, et al 2013 Structural and functional characterization of K339T substitution identified in the PB2 subunit cap-binding pocket of influenza A virus. J Biol Chem 288(16): 11013–11023. 10.1074/jbc.M112.392878 23436652 PMC3630847

