# Supplementary material for: Interaction between influenza A virus nucleoprotein and PB2 cap-binding domain is mediated by RNA
Source: PLoS One. 2020 Sep 28;15(9):e0239899. doi: 10.1371/journal.pone.0239899 (PMC7521707; doi:10.1371/journal.pone.0239899)

Figure 1A & B. Western blot image captured by chemiluminescence detection on film.

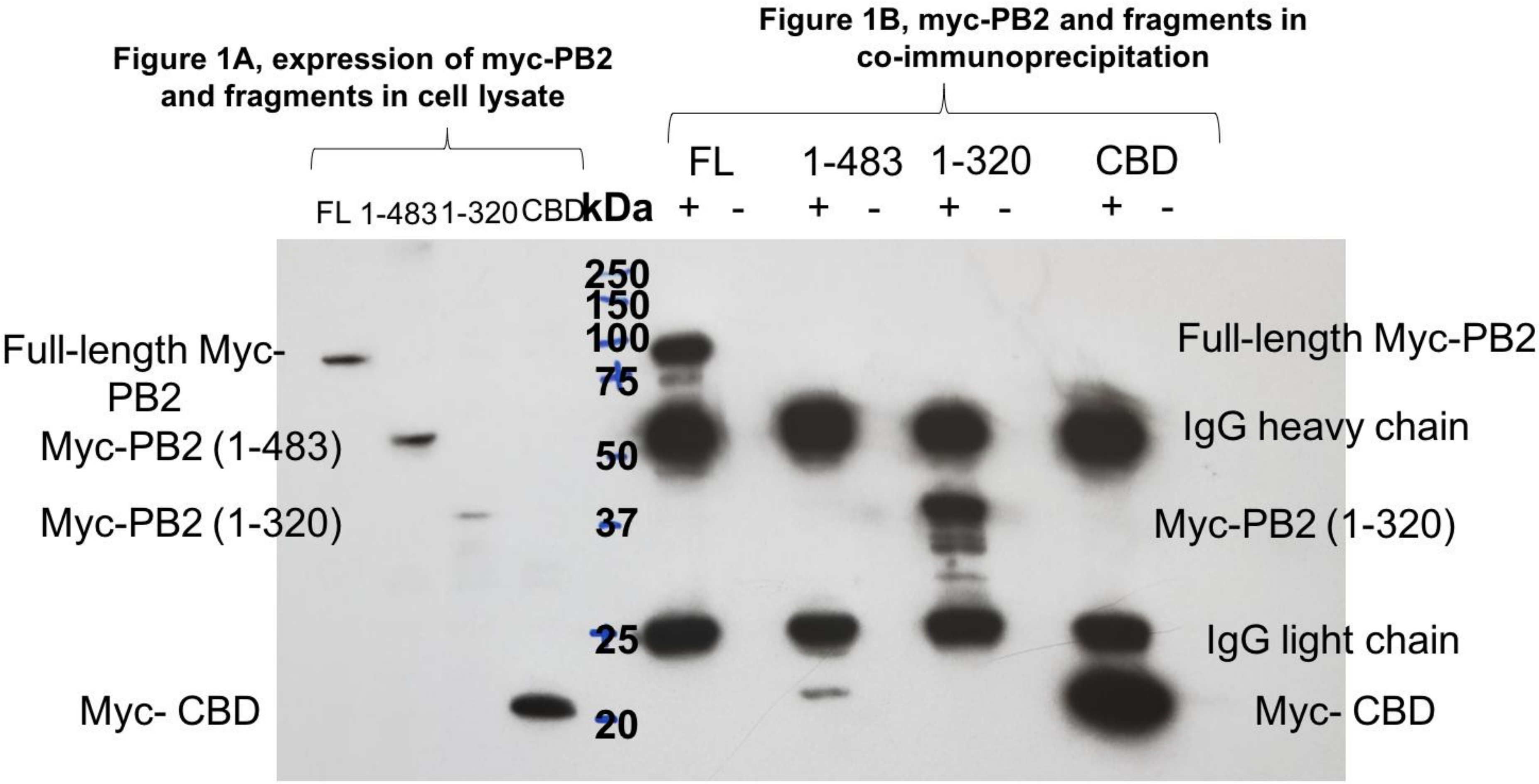

Repeat of figure 1A & B. Western blot image captured by chemiluminescence detection on film.

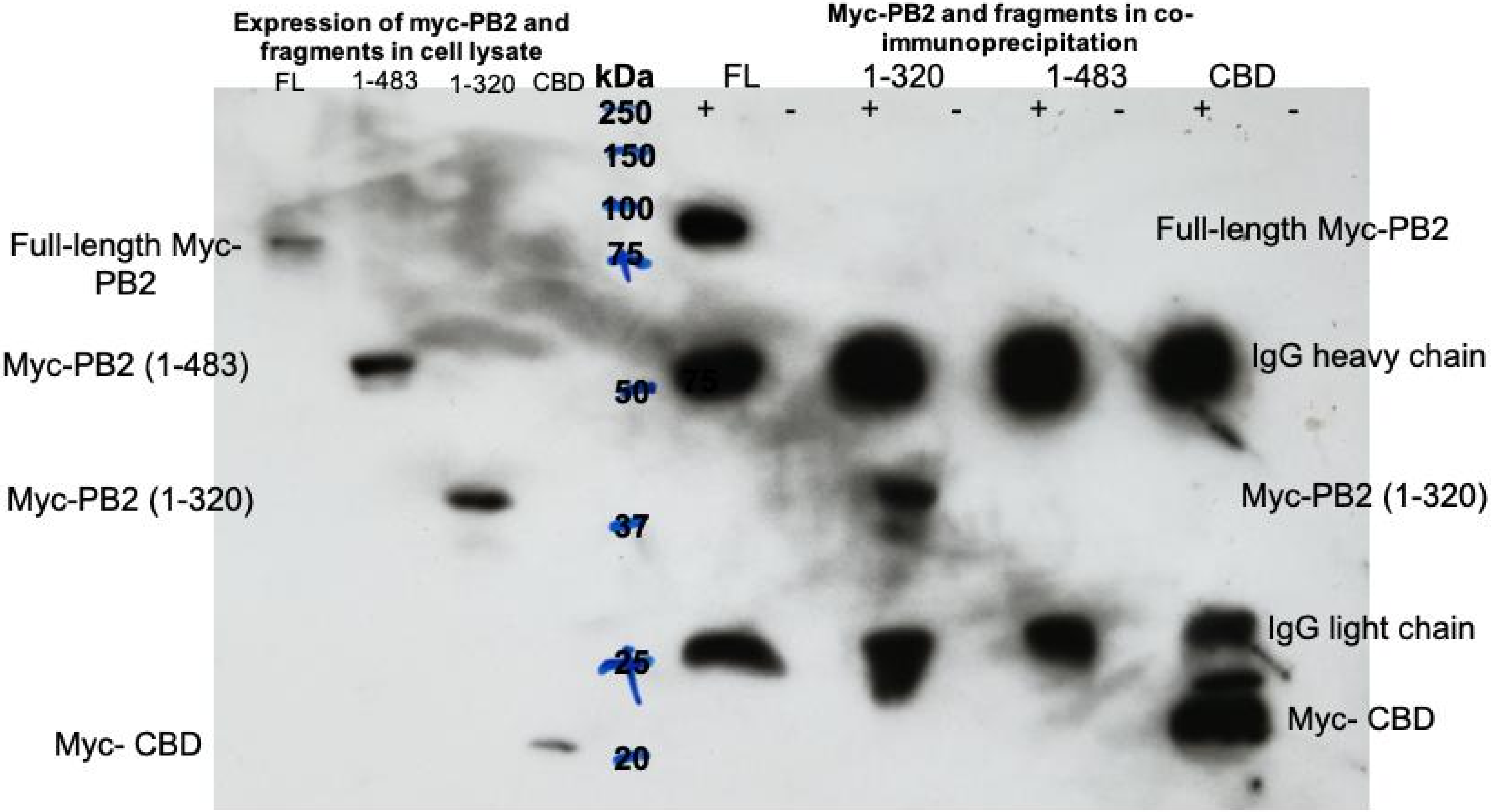

Figure 1A & B. Western blot image captured by chemiluminescence detection on film.

Figure 1A, expression of NP in cell lysate

Figure 1B, NP in co-immunoprecipitation

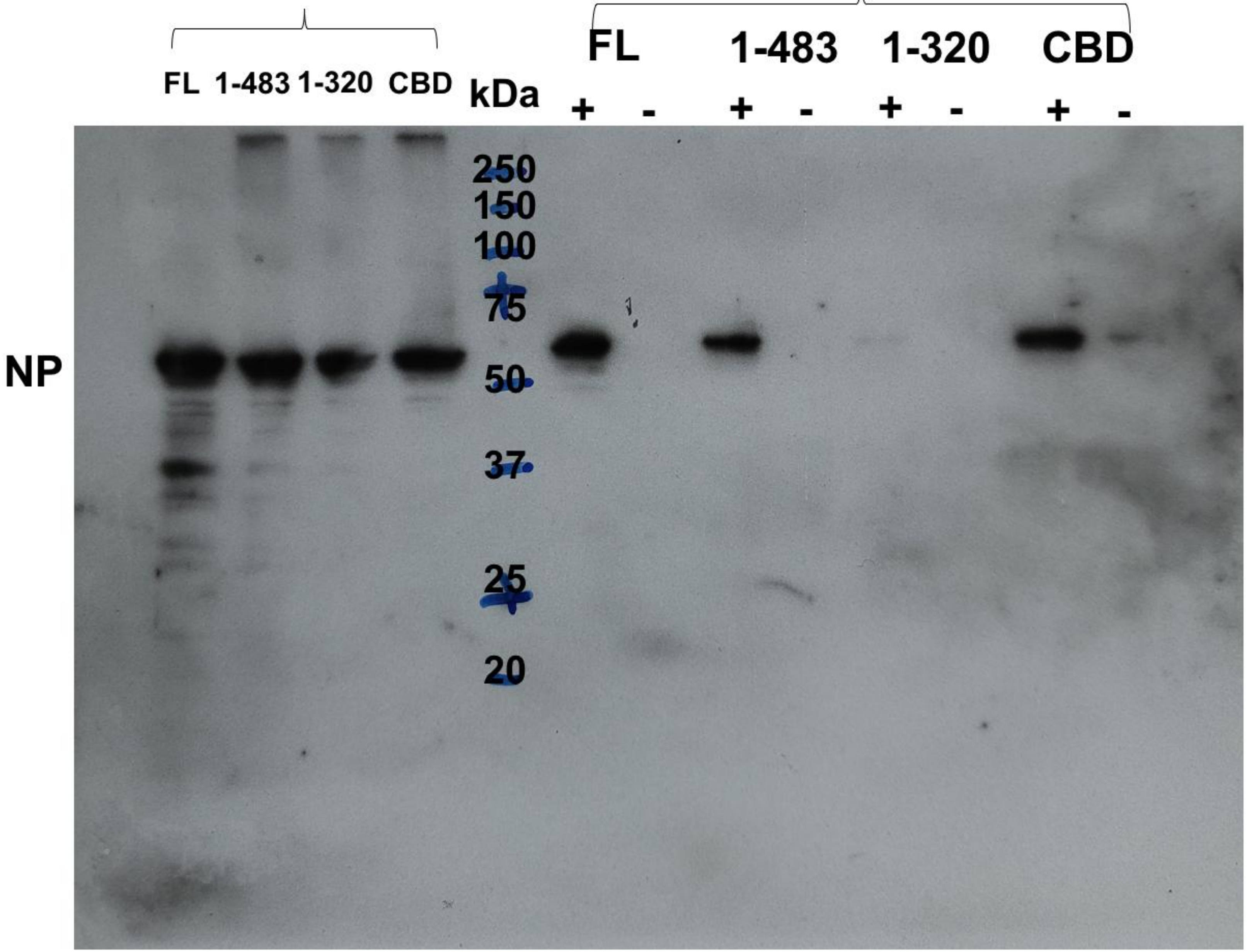

Repeat of figure 1A & B. Western blot image captured by chemiluminescence detection on film.

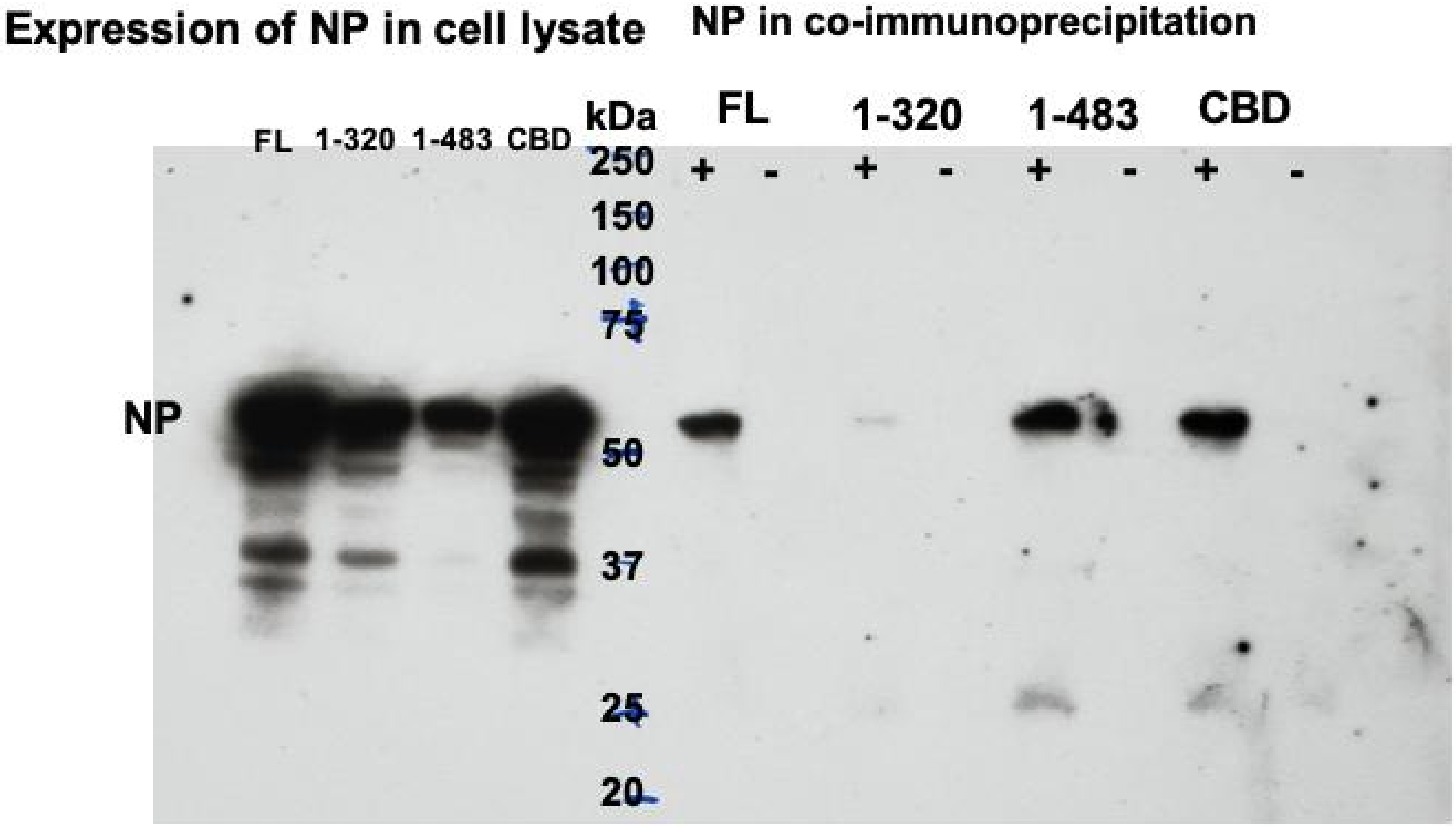

Figure 2A. Expression of NP and myc-CBD in lysate. Western blot image captured by chemiluminescence detection on film.

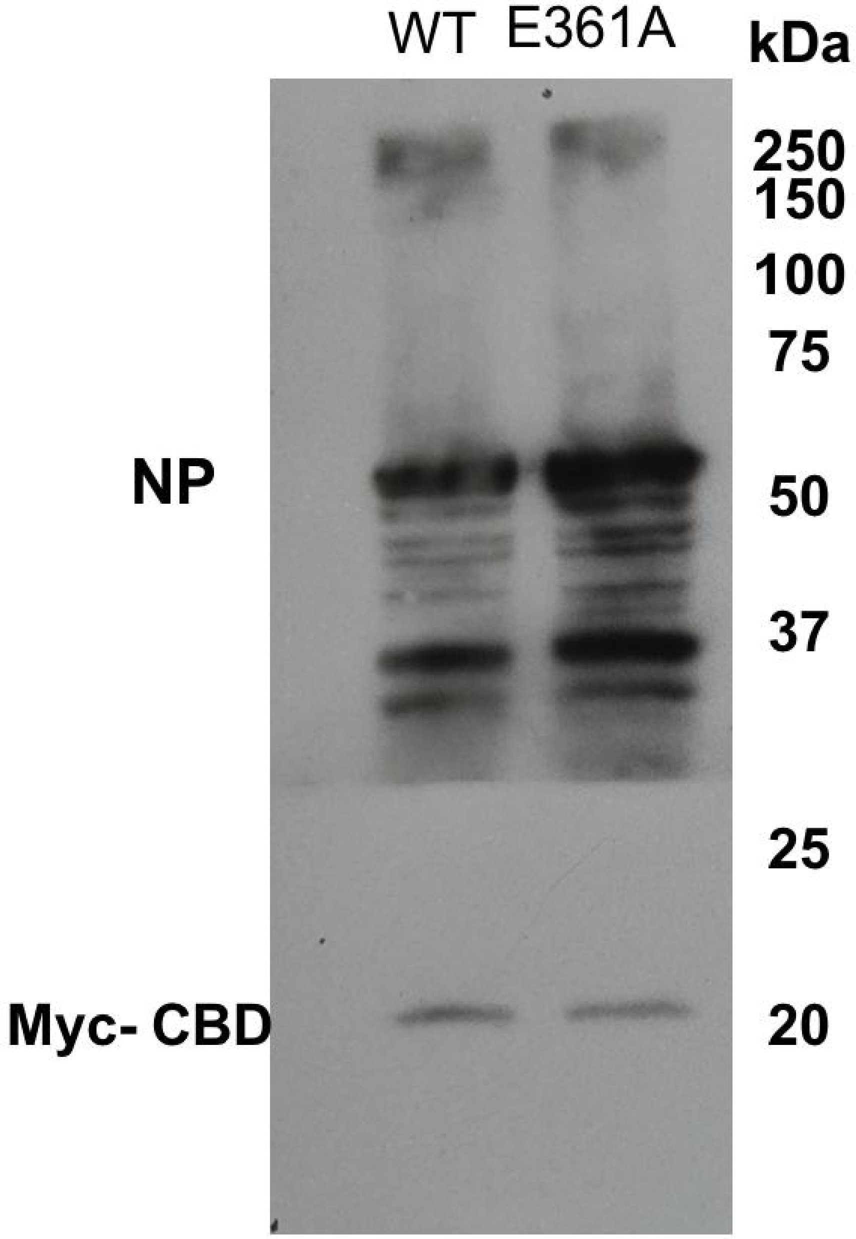

Figure 2B. Western blot image captured by chemiluminescence detection on film.

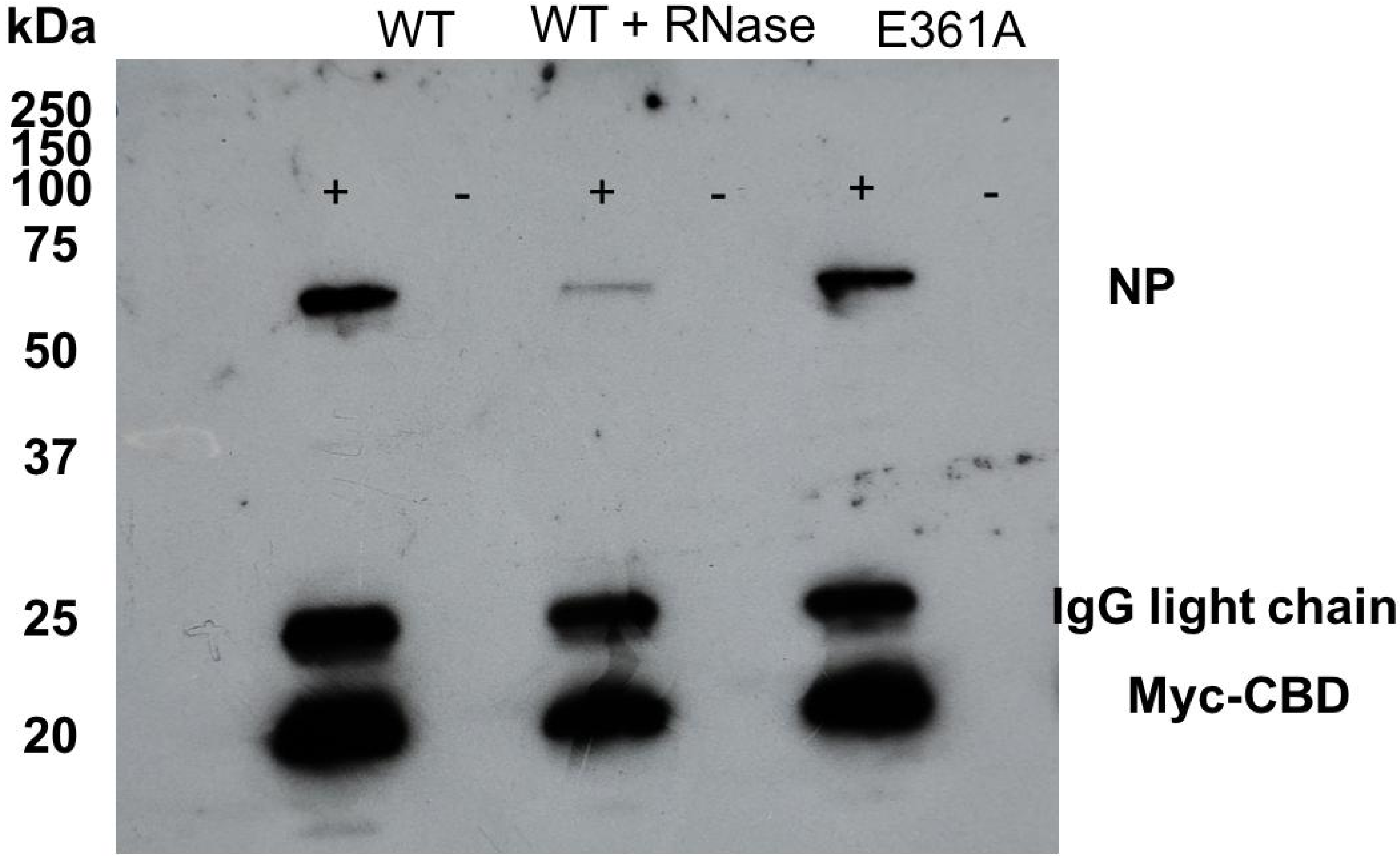

Figure 2C. 12% SDS-PAGE of pull down assay. Proteins in wash and elute fractions were detected by coomassie blue staining. Image was captured by Gel Doc imager (Bio-Rad)

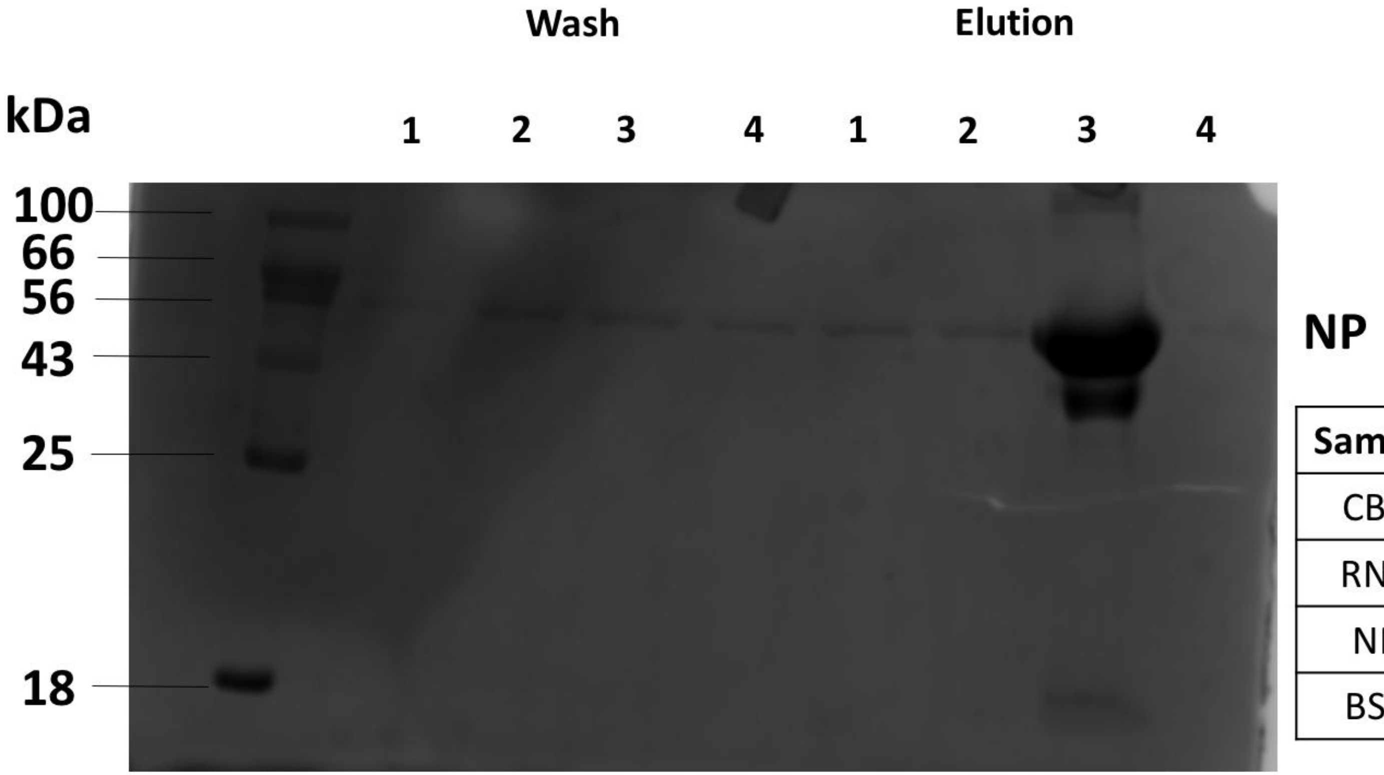

Sample	1	2	3	4
CBD	+	-	+	-
RNA	-	+	+	+
NP	+	+	+	+
BSA	-	-	-	+

Figure 3B. Expression of PB2 and variants. Western blot image captured by chemiluminescence detection on film.

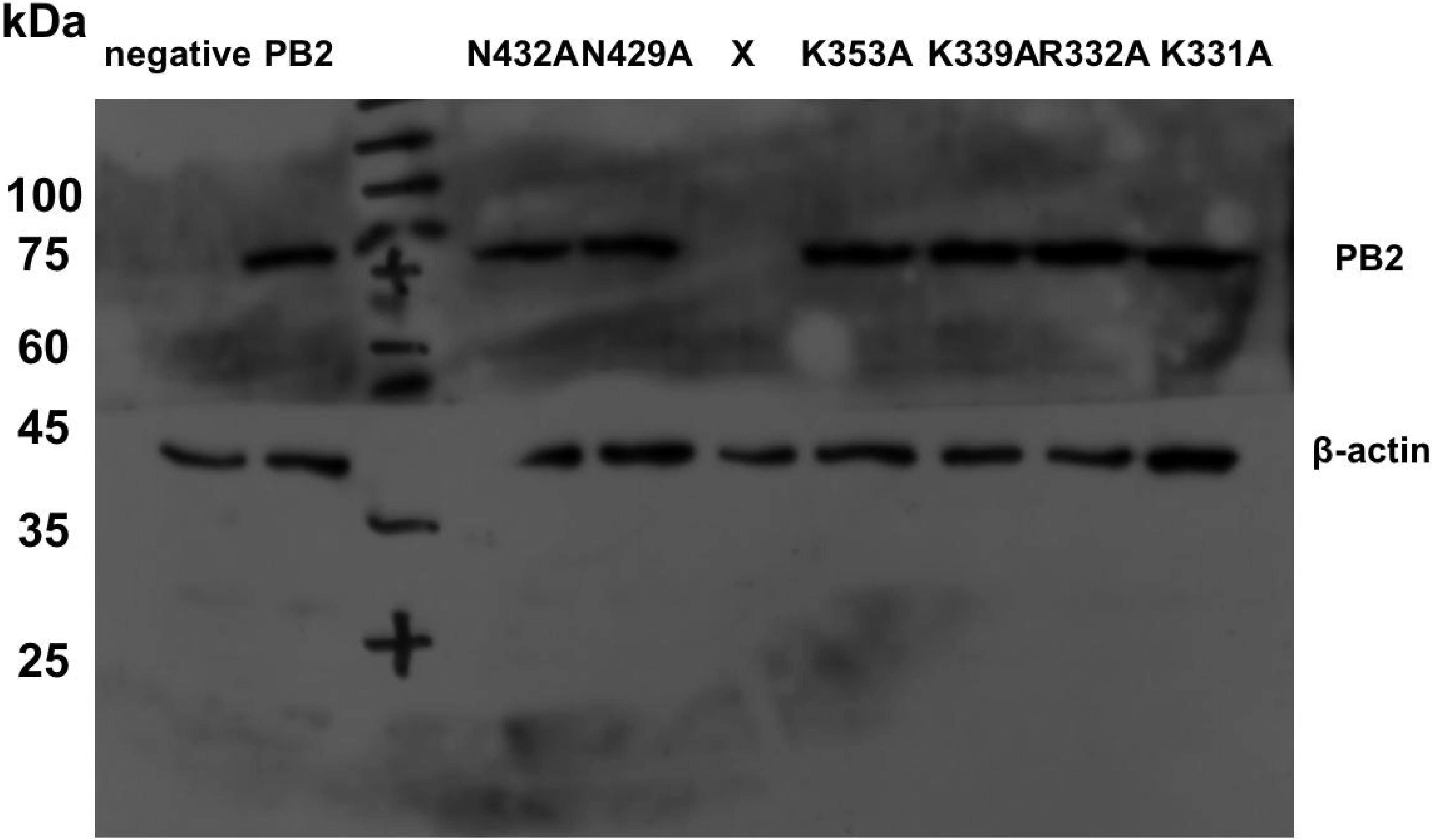

Figure 3C. Western blot image captured by chemiluminescence detection on film.

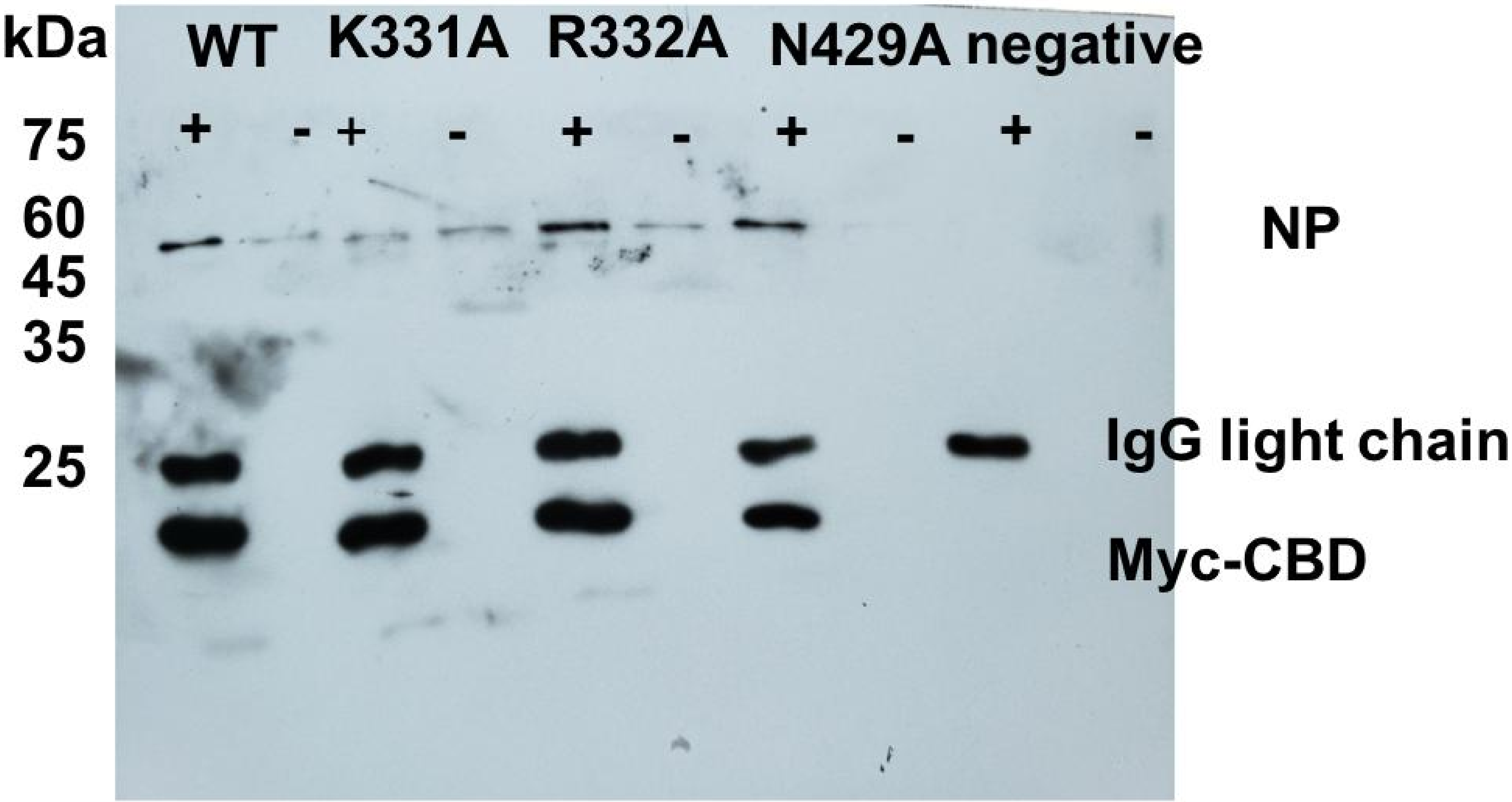

Figure 3C. Expression of NP and myc-CBD. Western blot image captured by chemiluminescence detection on film.

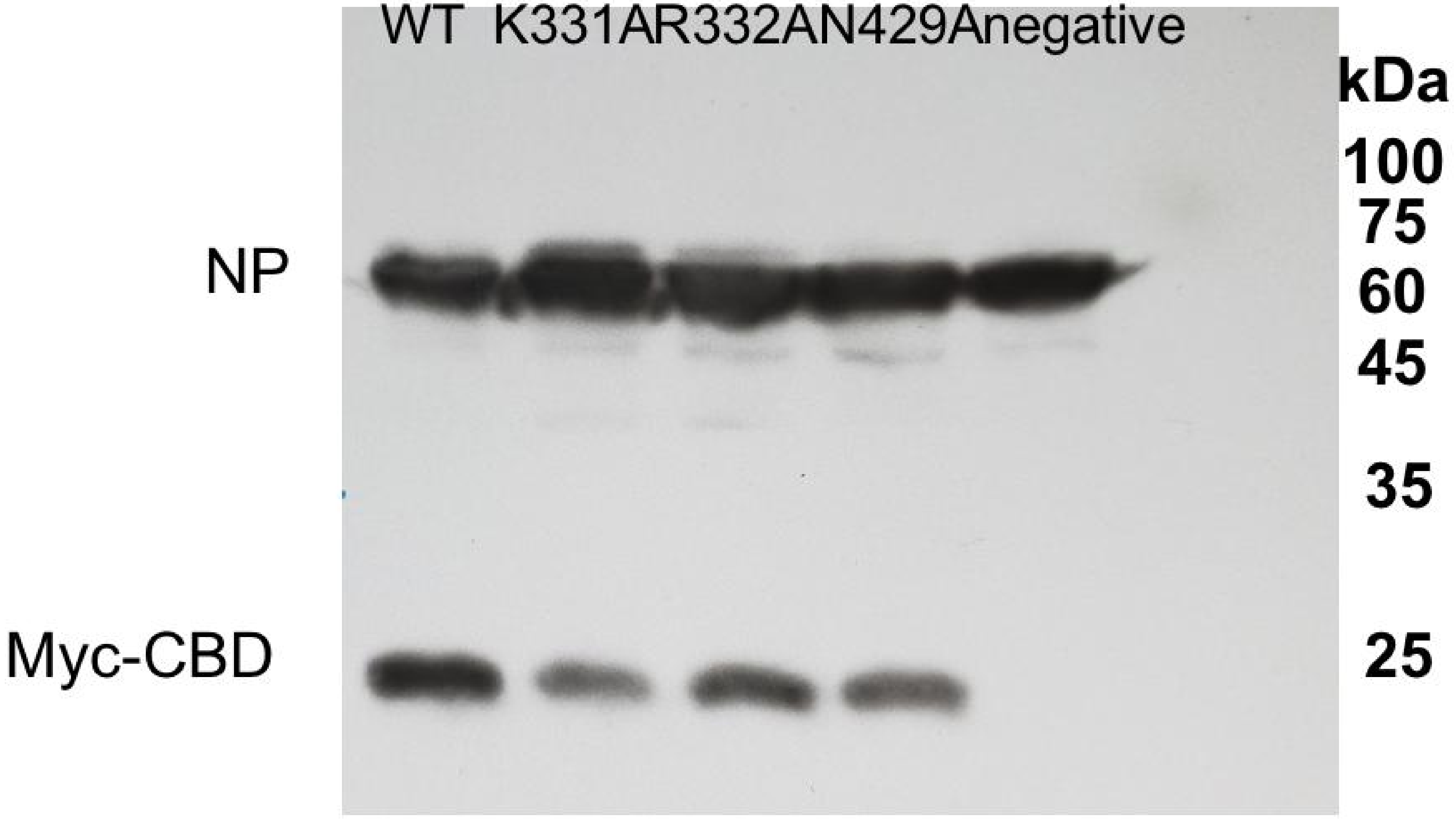

Figure 4A. Western blot image captured by chemiluminescence detection on film.

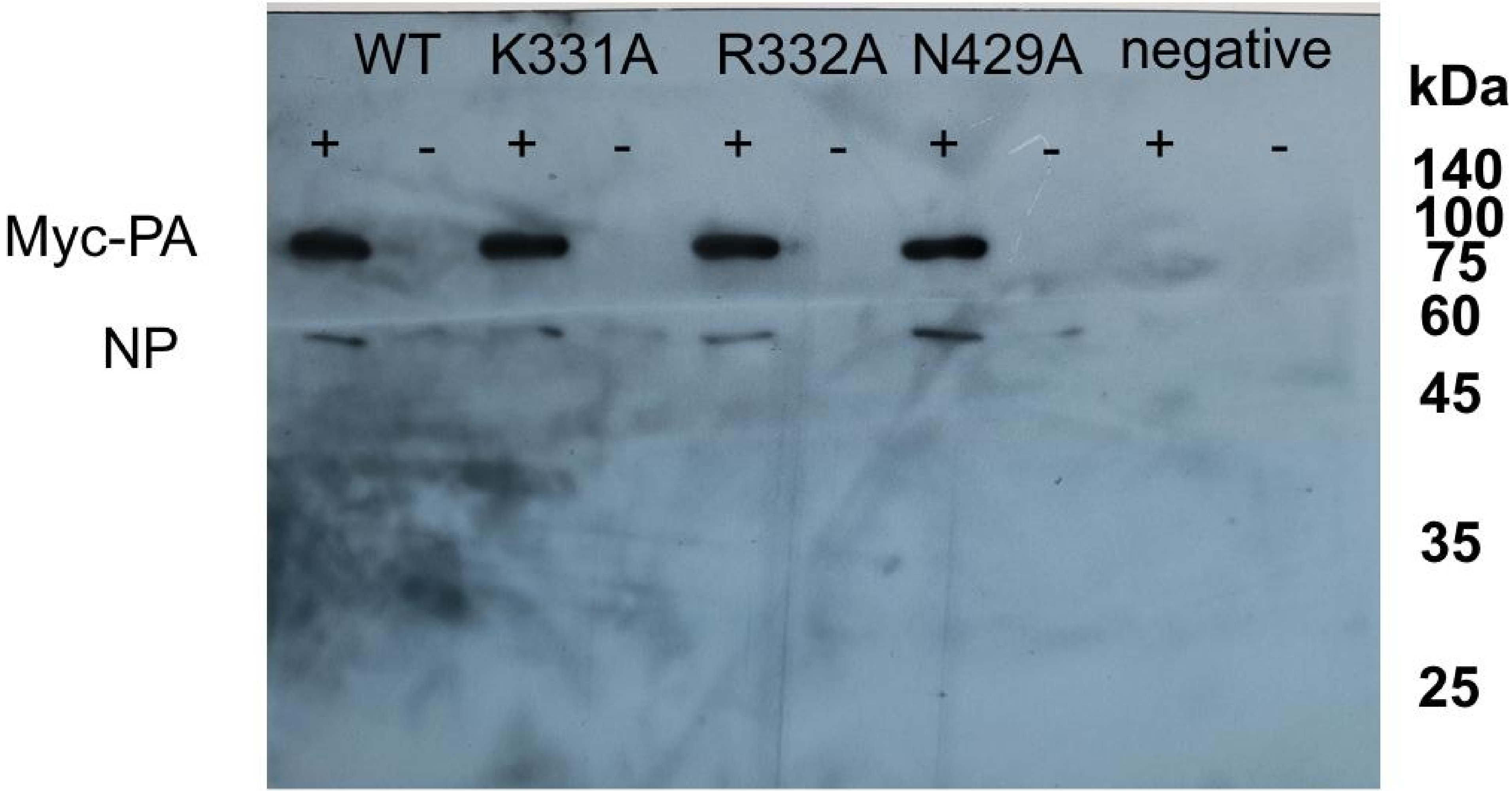

Figure 4B. Expression of Myc-PA. Western blot image captured by chemiluminescence detection on film.

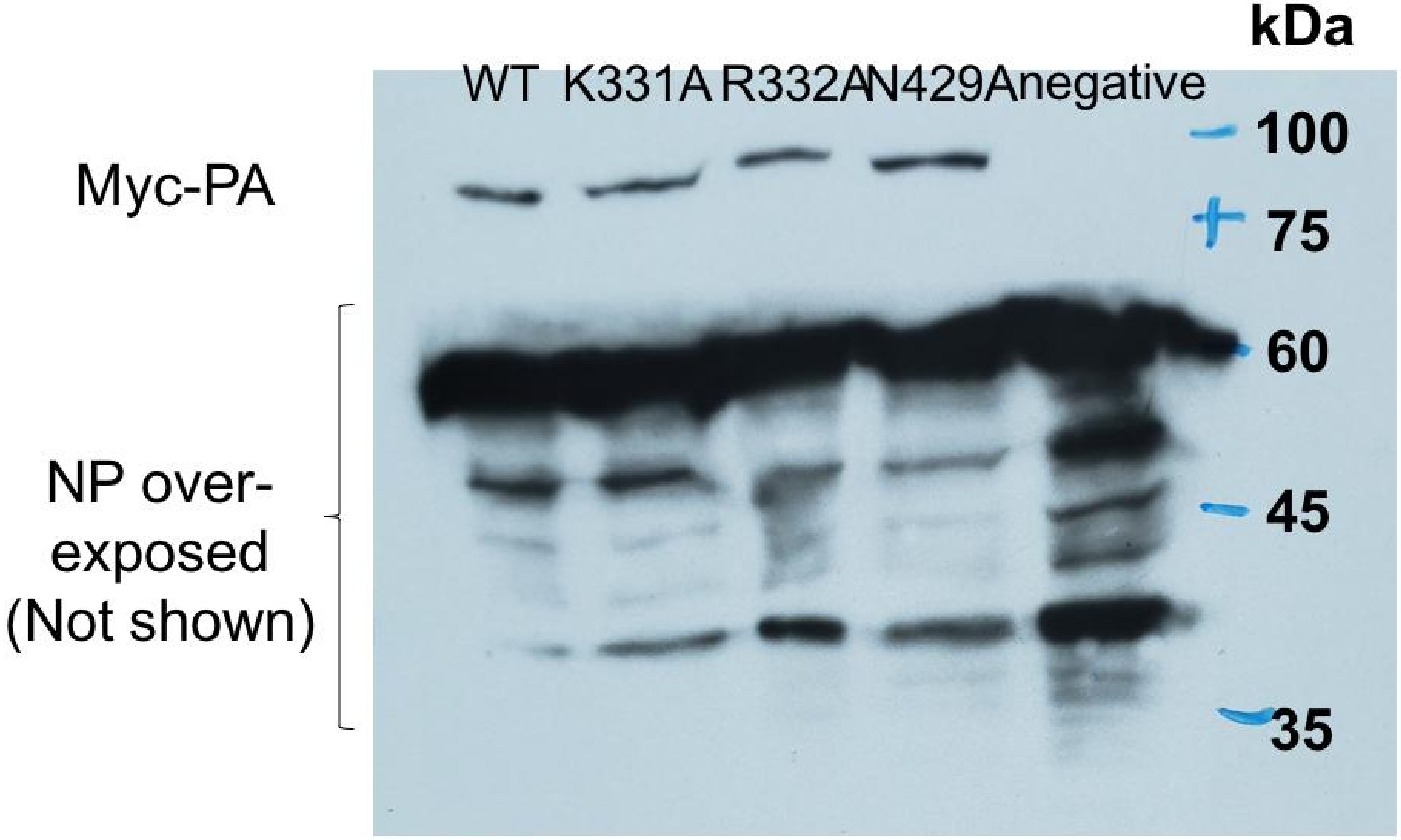

Figure 4B. Expression of PB2. Western blot image captured by chemiluminescence detection on film.

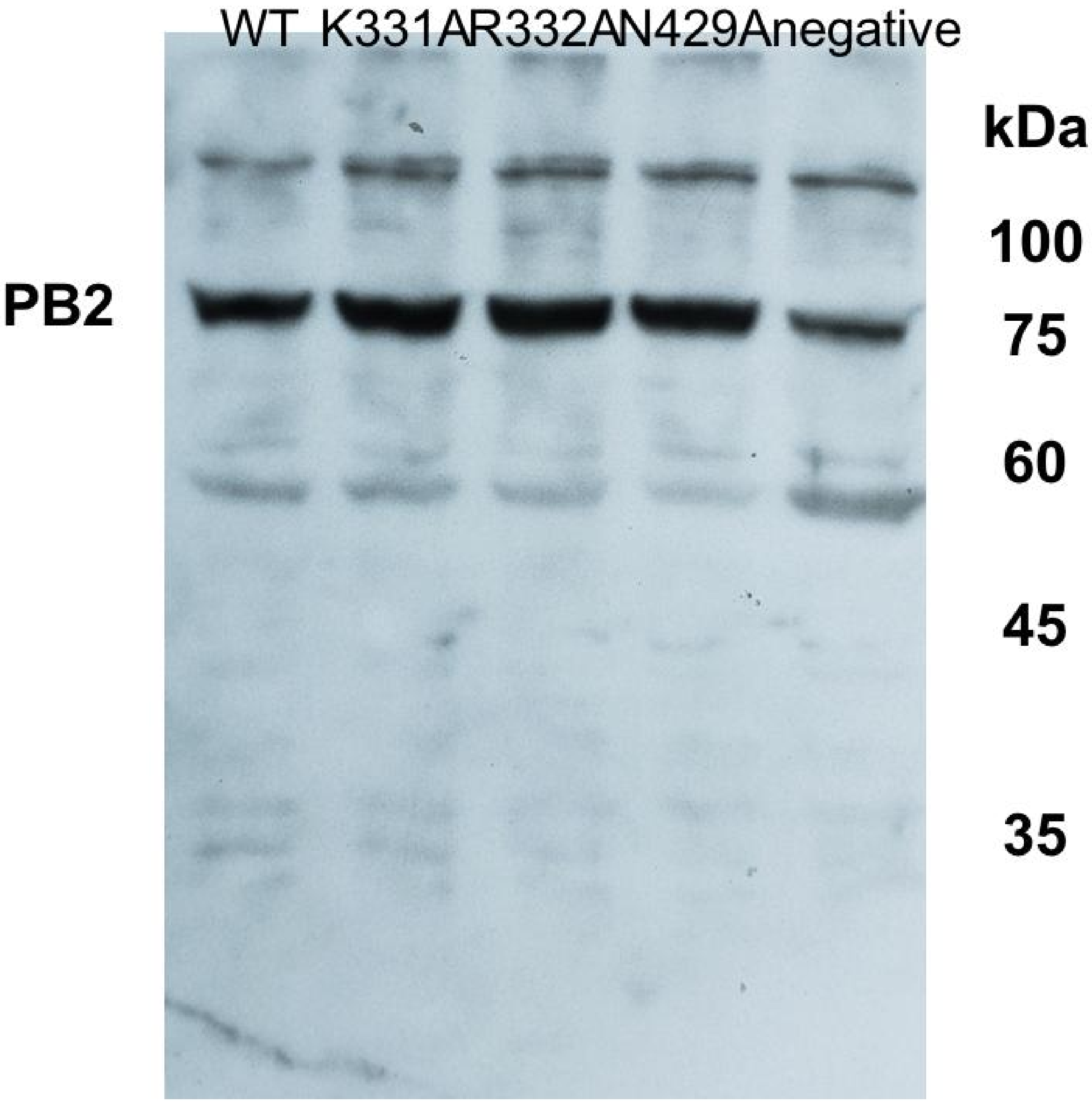

Figure 4B. Expression of PB1. Western blot image captured by chemiluminescence detection on film.

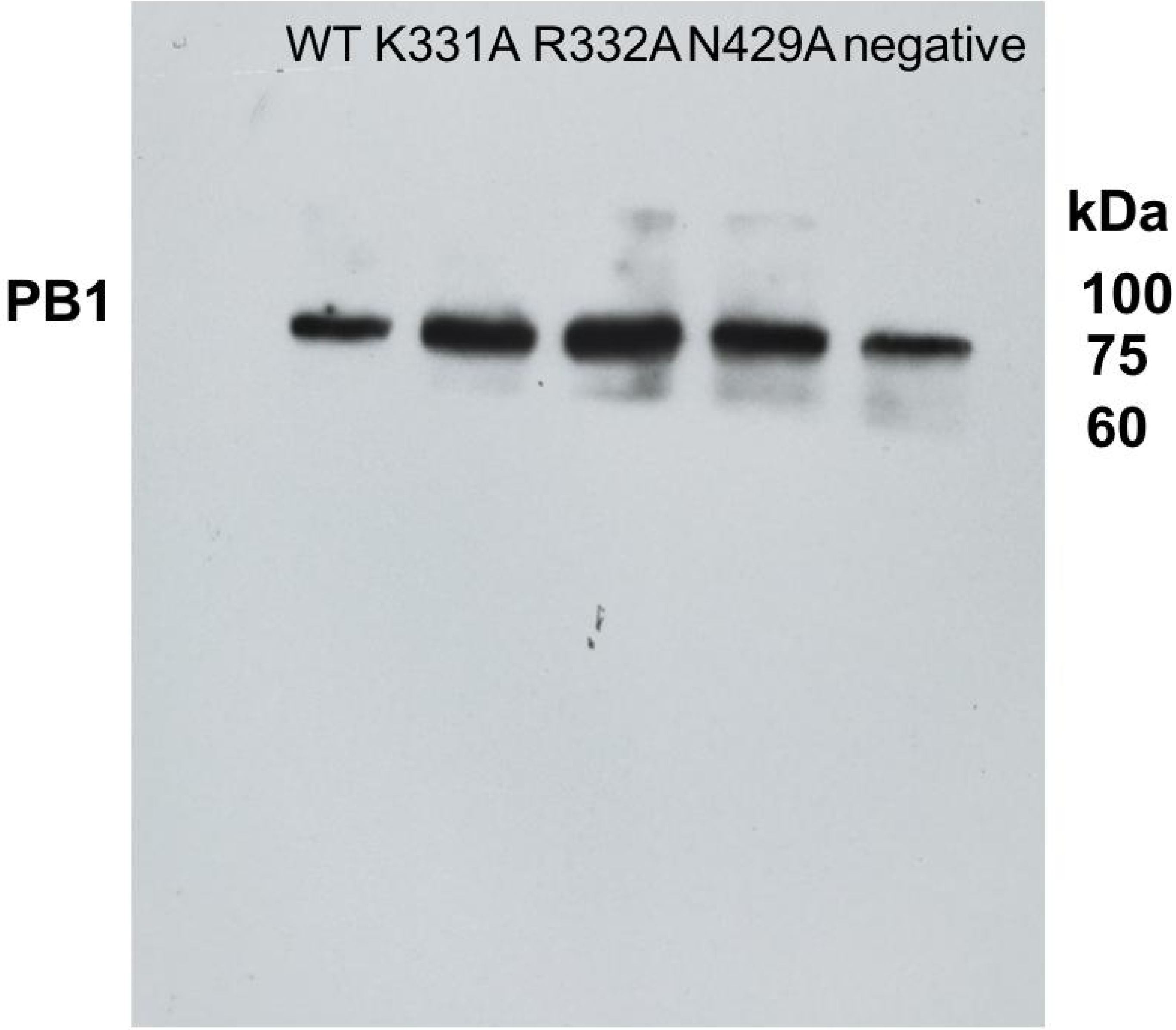

Figure 4B. Expression of NP. Western blot image captured by chemiluminescence detection on film.

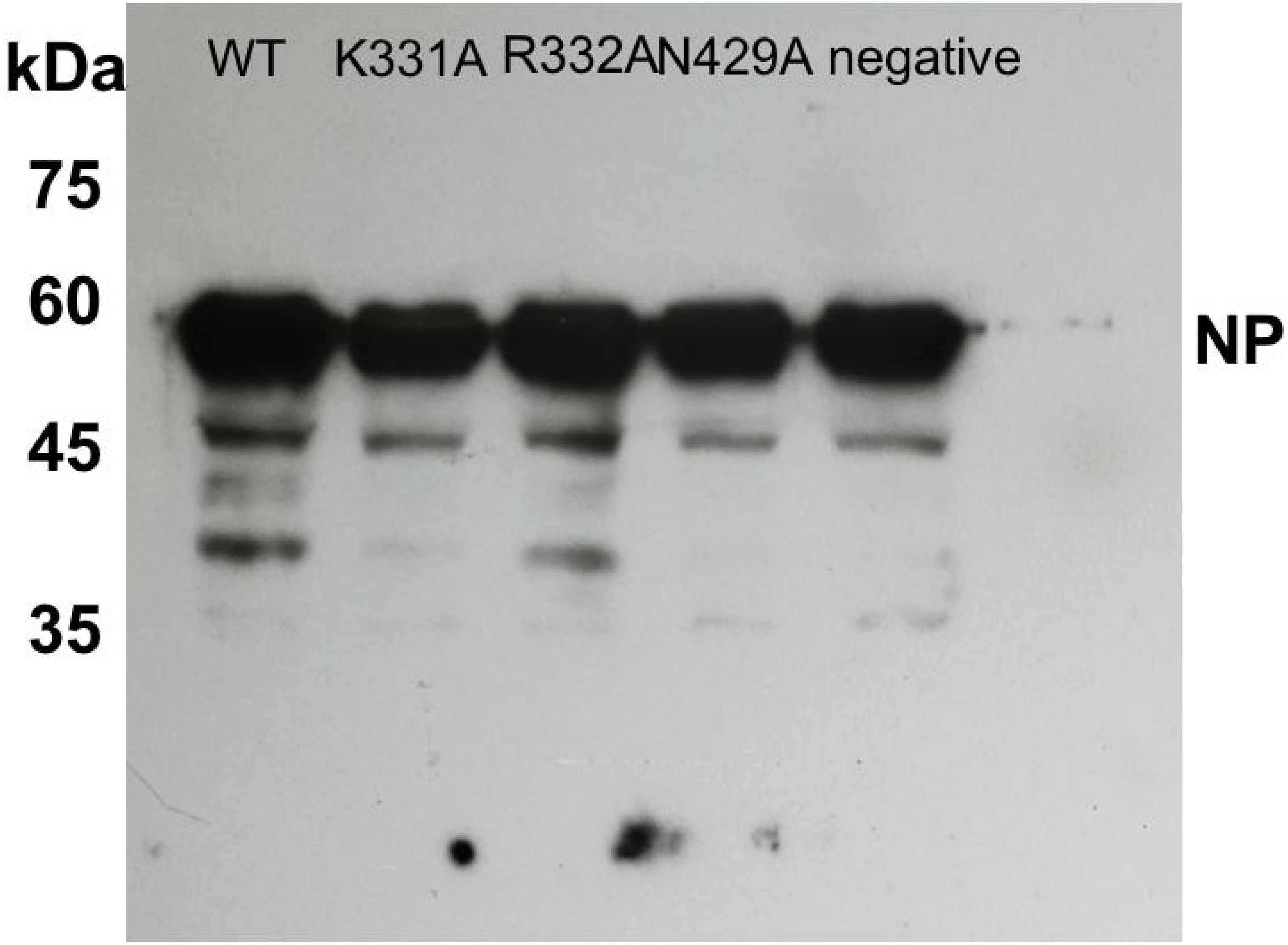

Supplement: S1 File — (PDF) [file pone.0239899.s001.pdf]
