# Supplementary material for: Interaction between influenza A virus nucleoprotein and PB2 cap-binding domain is mediated by RNA
Source: PLoS One. 2020 Sep 28;15(9):e0239899. doi: 10.1371/journal.pone.0239899 (PMC7521707; doi:10.1371/journal.pone.0239899)

S1 Figure. Myc-tagged NP detected by monoclonal anti-myc antibody (Cell Signaling) or anti-NP serum.Western blot image captured by chemiluminescence detection on film.

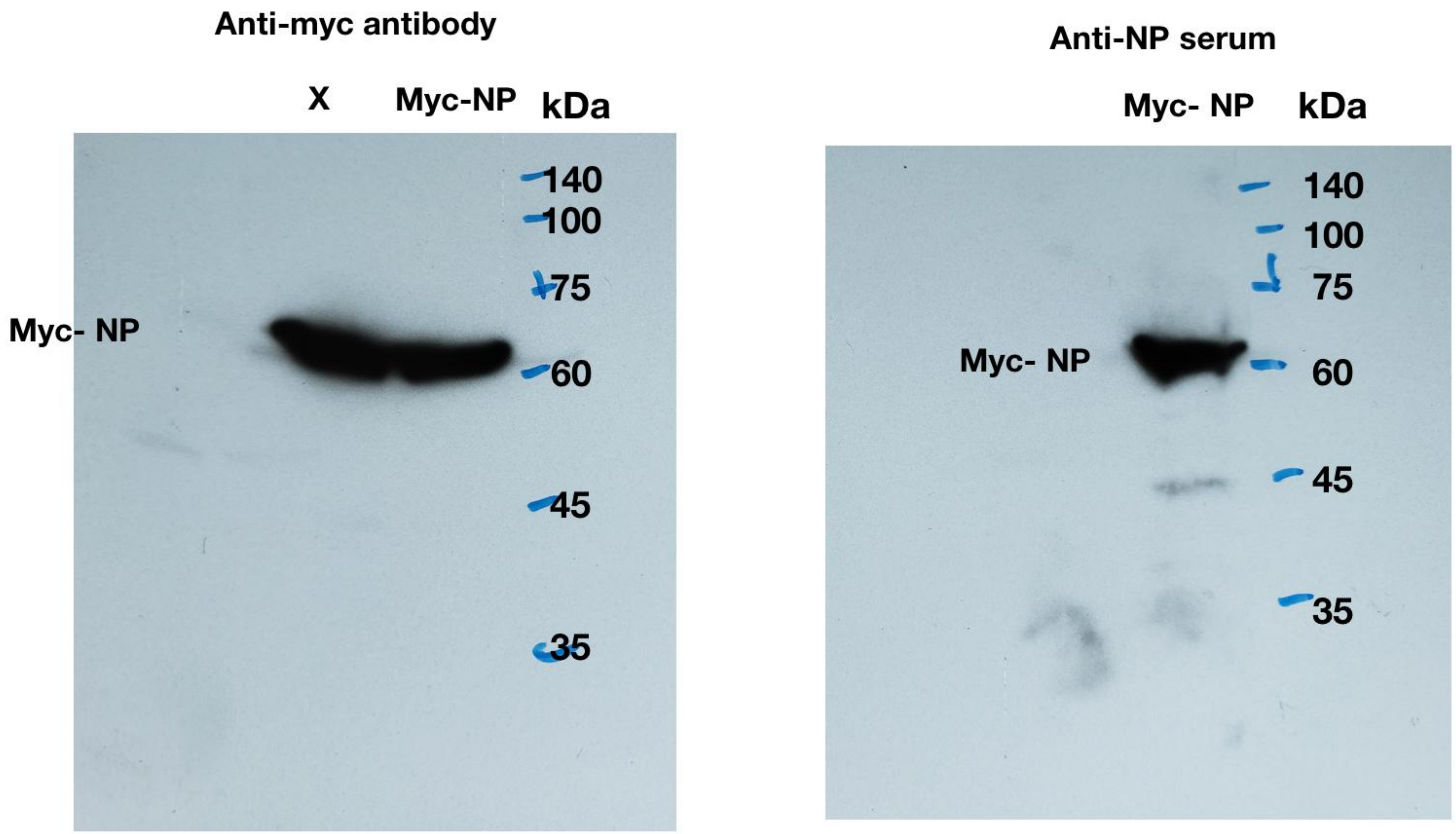

Supplement: S1 Fig — Plasmid encoding myc-tagged NP was transfected into HEK 293 cells, cell lysate was obtained after 48 hour incubation. Expressed proteins were detected by anti-myc monoclonal antibody and anti-NP serum respectively by Western blot. (PDF) [file pone.0239899.s002.pdf]
